# The little brain and the seahorse: Cerebellar-hippocampal interactions

**DOI:** 10.3389/fnsys.2023.1158492

**Published:** 2023-03-23

**Authors:** Jessica M. Froula, Shayne D. Hastings, Esther Krook-Magnuson

**Affiliations:** Department of Neuroscience, University of Minnesota, Minneapolis, MN, United States

**Keywords:** coherence, oscillations, fastigial, supramammillary, place cells, navigation, vermis, social

## Abstract

There is a growing appreciation for the cerebellum beyond its role in motor function and accumulating evidence that the cerebellum and hippocampus interact across a range of brain states and behaviors. Acute and chronic manipulations, simultaneous recordings, and imaging studies together indicate coordinated coactivation and a bidirectional functional connectivity relevant for various physiological functions, including spatiotemporal processing. This bidirectional functional connectivity is likely supported by multiple circuit paths. It is also important in temporal lobe epilepsy: the cerebellum is impacted by seizures and epilepsy, and modulation of cerebellar circuitry can be an effective strategy to inhibit hippocampal seizures. This review highlights some of the recent key hippobellum literature.

## Introduction

While classically considered a motor structure, there is a growing appreciation for the larger roles the cerebellum can play. One relatively understudied area that nicely highlights the potential cognitive roles of the cerebellum is what our lab has termed the ‘hippobellum’ – namely, a bidirectional functional connectivity between the cerebellum and the hippocampus. In contrast to the cerebellum, the hippocampus is chiefly considered a cognitive structure, well known for its roles in learning, memory, and spatial processing. A relatively small, but growing, body of literature supports bidirectional functional connectivity between the hippocampus and the cerebellum, in both healthy brains and in pathological conditions, such as epilepsy. Research is beginning to elucidate potential underlying anatomical pathways, although more studies are needed. In this review, we highlight some of the key hippobellum literature and discuss potential insights and clinical avenues their connection provides.

## The complicated path to a hippobellum circuit (anatomical underpinnings)

Seminal tracing studies in primates examining cerebellar connections to the primary motor cortex uncovered a now canonical loop: (1) cerebellar nuclear neurons (DCN) project to motor thalamus, (2) motor thalamus projects to motor cortex, (3) motor cortex projects to pontine nuclei, (4) pontine nuclei project to cerebellar cortex via mossy fibers (with collaterals to the DCN), and (5) cerebellar cortex projects to DCN (Allen and Tsukahara, 1974; Orioli and Strick, 1989). Further studies of cerebellar outputs showed similar connectivity loops with a variety of neocortical areas, supporting both motor and non-motor functions (Middleton and Strick, 1998; Kelly and Strick, 2003; Bostan et al., 2013; Caligiore et al., 2017). Notably, none of these represent direct projections from the cerebellum to the neocortex and back; they are all multi-synaptic (Kelly and Strick, 2003). While perhaps obvious, it is important to state that a connection between two structures does not need to be direct to be a powerful mediator of brain function.

In contrast to these now canonical loops, the anatomical underpinnings of cerebellar-hippocampal communication is not yet firmly established. Similar to cerebellar-neocortical connections, which occur via one or more intermediary regions, there is almost certainly one or more intermediary structures between the cerebellum and the hippocampus (Yu and Krook-Magnuson, 2015; Watson et al., 2019; Fujita et al., 2020). In this regard, cerebellar influences on the hippocampus is not unique. However, the intermediary structures at play appear to be different for the allocortex versus neocortex, as discussed more below. As an important side note – in the past, a direct connection between the structures was argued based on lesion studies and the timing of electrical responses (Heath and Harper, 1974; Snider and Maiti, 1976; Heath et al., 1978; Newman and Reza, 1979). However, improved techniques have refuted this idea (Badura et al., 2018; Krook-Magnuson, 2019; Watson et al., 2019; Fujita et al., 2020), at least in rodents (Liu et al., 2012; Arrigo et al., 2014), and suggest instead a multi-synaptic pathway.

**FIGURE 1:**
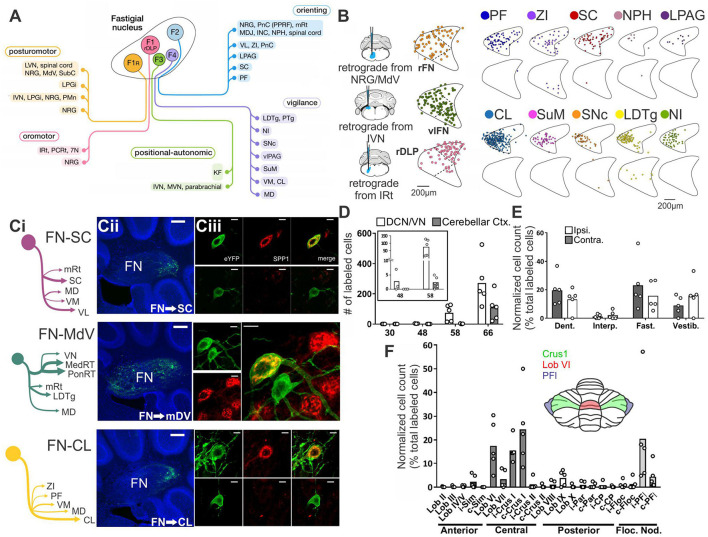
There are many avenues by which the cerebellum could influence the hippocampus. **(A)** Diagram modified from Fujita et al. (2020) outlining organized outputs from the Fastigial Nucleus (FN). FN outputs project to a wide variety of both motor and non-motor regions via segregated output channels. The sheer number of outputs from a single output nucleus of the cerebellum illustrates the complexity of determining which targets may serve as intermediaries for a hippobellum circuit. **(B)** FN neurons projecting to different downstream areas – labeled via retrograde tracer injections (left) into target regions – reside in distinct, but overlapping, FN subregions (i.e., rostral, ventral, dorsolateral, and caudal). Dots within the FN outlines are individual neurons that project to each output structure. The schematics on the right show two coronal outlines of the fastigial nucleus – one caudal (top rows) and one rostral (bottom rows). Note that several downstream regions are targeted by neurons residing largely in the caudal portion of the FN. **(C)** A detailed study of three output channels of the FN further illustrates that while FN neurons project to multiple areas (i.e., have collaterals), they do so in a structured manner, with collaterals to specific subsets of target regions. Retrograde-AAV-Cre was injected into a target region (SC, MdV, or CL), and a Cre-dependent AAV was injected into the FN, allowing visualization of projections from specific FN neuronal populations. **(Ci)** Arrow thickness indicates density of each projection relative to general FN input to that region, for neurons labeled based on their projections to the SC (top), the MdV (middle), or the CL (bottom). **(Cii)** FN cells retrogradely labeled from injections in the SC (top), MdV (middle), or CL (bottom) are in distinct but overlapping areas of the FN. Scale bars: 200 μm. Images show a sagittal view of the FN; note the clustering of both FN-SC and FN-CL neurons in the more caudal portions. **(Ciii)** Projection neurons have a heterogeneous molecular profile, with both SPP1 + (red and green overlay) and SPP1- (green only) FN cells projecting to each examined extra-cerebellar region. Scale bars: 10 μm. **(D–F)** Retrograde multi-synaptic rabies (RABV) tracing following injection into the dorsal dentate gyrus shows that multiple cerebellar areas are anatomically, multi-synaptically, connected to the hippocampus. Each dot represents one animal. **(D)** Number of labeled cells at each time point post RABV injection. A lack of labeled cells in the cerebellum at the 30-h time point suggests there is no monosynaptic connection between the cerebellum and the hippocampus. A small number of cells were labeled in the cerebellum after 48 h (graph inset) indicating a possible disynaptic connection, but most labeled cells were seen at 58 h and 66 h post injection, suggesting more complicated multi-synaptic circuity. **(E)** Distribution of labeled cells in each cerebellar nucleus at the 58-h time point. Most RABV labeled cells were found in the dentate, fastigial, and vestibular nuclei providing multiple routes for cerebello-hippocampal communication. **(F)** Distribution of cells in the cerebellar cortex. RABV + cells are concentrated in lobule VI in the vermis, and Crus I and paraflocculus in the hemispheres, highlighted in the inset schematic. Figure panels modified from Fujita et al. (2020) panels **(A,B)**, (Streng et al., 2021) panel **(C)**, and (Watson et al., 2019) panels **(D-F)**, all under a CC-BY 4.0 license. 7N, facial nucleus; CL, centrolateral thalamic nucleus; contra., c, contralateral; CP, copula; ctx., cortex; DCN, deep cerebellar nuclei; dent, dentate nucleus; DLP, dorsolateral protuberance of FN; rFN, rostral FN; vlFN, ventrolateral FN; Floc, flocculus; Floc. Nod., flocculonodular lobe; INC, interstitial nucleus of Cajal; IRt, intermediate reticular nucleus; interp., interposed nucleus; IVN, intermediate vestibular nucleus; ipsi., i, ipsilateral; KF, Kölliker-Fuse nucleus; LDTg, laterodorsal tegmental nucleus; LPAG, lateral periaqueductal gray; LPGi, lateral paragigantocellular nucleus; LVN, lateral vestibular nucleus; lob, lobule; MD, mediodorsal thalamic nucleus; MDJ, mesodiencephalic junction; MedRT, medullary reticular nucleus; MdV, medullary reticular nucleus, ventral; mRt, mesencephalic reticular formation; MVN, medial vestibular nucleus; NPH, nucleus prepositus hypoglossi; NI, nucleus incertus; NRG, nucleus reticularis gigantocellularis; PCRt, parvocellular reticular nucleus; PF, parafascicular thalamic nucleus; PFl, paraflocculus; i-Par, c-Par, paramedian lobule; PMn, paramedian reticular nucleus; PonRT, pontine reticular nucleus; PnC, pontine reticular nucleus, caudal; PPRF, paramedian pontine reticular formation; PTg, pedunculotegmental nucleus; Sim, simplex; SC, superior colliculus; SNc, substantia nigra, pars compacta; SubC, subcoeruleus nucleus; SuM, supramammillary region; VL, ventrolateral thalamic nucleus; vlPAG, ventrolateral periaqueductal gray; VM, ventromedial thalamic nucleus; vestib., VN, vestibular nuclei; ZI, zona incerta ZI.

Multi-synaptic connectivity provides for a wealth of potential hippobellum pathways. This provides both a richness – allowing function and substructure specific connectivity – and extra experimental challenges. Sophisticated circuit tracing methods are required to fill in the intermediate structures involved, and in turn the function(s) carried by each. Fortunately, tracing techniques are being continuously developed and refined and are increasingly used to probe functional connectivity. While new tools provide new opportunities to examine cerebellar-hippocampal and hippocampal-cerebellar connectivity, the question of pathways remains excruciatingly difficult and complex. This is in part due to the large number of brain regions the cerebellum does directly project to. Fujita and colleagues, for example, found that the FN alone has over 60 (direct) extra-cerebellar targets (Figures 1A, B; Fujita et al., 2020). We will not attempt a full review of inputs and outputs of the cerebellum and the hippocampus. For extensive recent reviews of cerebellar outputs, see (Kang et al., 2021; Novello et al., 2022); for reviews of hippocampal inputs and outputs see (Atoji and Wild, 2006; van Strien et al., 2009). Instead, we will highlight some recent research that is particularly relevant with regards to potential hippobellum connectivity.

In addition to the sheer number of FN targets identified by Fujita et al. (2020), they also found that outputs from the Fastigial Nucleus are organized into output channels, with different FN neurons (often, but not always, residing in different portions of the FN, and often, but not always, displaying different gene expression profiles) projecting to different groups of downstream neurons (Figure 1B). Some of these output channels have been further refined in our recent work (Figure 1C) and examined in the context of the hippobellum in epilepsy (discussed later in this review). Notably, none of the over sixty downstream targets identified by Fujita and colleagues was the hippocampus (supporting a lack of direct connection between the two structures). Some areas receiving direct input from the FN that are of potential interest in the context of the hippobellum include the nucleus incertus and the supramammillary nucleus (SuM) – which both have direct projections to the hippocampus (nucleus incertus projections: Goto et al., 2001; Olucha-Bordonau et al., 2003; Szõnyi et al., 2019; SuM projections: Wyss et al., 1979; Haglund et al., 1984) – as well as the superior colliculus, reticular formation, and thalamus, which were investigated for potential roles in the hippobellum in epilepsy (Streng et al., 2021).

Fujita and colleagues also used high titer AAV1 to express Cre in a reporter mouse line to examine multi-synaptic connectivity with the FN (Fujita et al., 2020). High titer AAV1 can travel transynaptically (typically only one synapse, though multi-synaptic travel has been occasionally observed) in antero- and retrograde directions (Zingg et al., 2017, 2020). This theoretically allowed identification of regions which receive disynaptic input from the FN, as cells (and, importantly, their terminals) which receive input from the FN would express fluorescent tdTomato protein in a cre-dependent manner. Thus, areas receiving input from neurons that receive input from the fastigial nucleus would express the red reporter protein. Interestingly, even with this approach, fibers in the hippocampus were not observed, suggesting that either the AAV1 approach taken did not capture all disynaptic outputs, or that there is not a disynaptic pathway connecting the fastigial nucleus to the hippocampus. Areas which had labeled fibers with this AAV1 approach, and thus which in turn could potentially mediate a multi-synaptic pathway to the hippocampus, include, but were not limited to, the medial septum and diagonal band, the striatum (possibly in part via the central lateral thalamus), and the cingulate, insular, and prelimbic cortices (though not the medial orbital or infralimbic cortices) (Fujita et al., 2020). Interestingly, no major labeling was noted in the amygdala (for monosynaptic or disynaptic labeling), suggesting this may not be a major route from the fastigial to the hippocampus (Fujita et al., 2020).

Of course, the fastigial nucleus is not the only output from the cerebellum. In addition to the other deep cerebellar nuclei (DCN), cerebellar output could be routed via the vestibular nuclei (Kang et al., 2021; Novello et al., 2022), the locus coeruleus (Schwarz et al., 2015), or the parabrachial nucleus (Hashimoto et al., 2018; Chen et al., 2022). There are a number of excellent reviews considering the relevance of the vestibular system to hippocampal processing (Smith, 1997; Smith et al., 2005; Hitier et al., 2014; Aitken et al., 2018), and the locus coeruleus has direct projections to the hippocampus (Loughlin et al., 1986; Klukowski and Harley, 1994; Kempadoo et al., 2016; Kaufman et al., 2020). It remains to be seen if the particular neurons in these areas which receive direct inputs from the DCN (or from Purkinje cells) in turn project to the hippocampus.

Another important study examining potential circuitry from the cerebellum to the hippocampus was done by Watson and colleagues, who used non-modified rabies virus with time post-injection as a proxy for the number of synapses travelled (Watson et al., 2019). Rabies was injected primarily into the dorsal dentate gyrus and labeled cells were examined at post-injection times of 30 (presumed monosynaptic), 48 (presumed disynaptic), 58 (presumed trisynaptic), and 66 h (presumed to be 4 synapses back) (Figure 1D). No rabies-labeled cerebellar cells were found at 30 h, again arguing against a direct connection. As expected, areas which did show labeling at 30 h included regions such as medial septum/diagonal band, SuM, and entorhinal cortices (Watson et al., 2019). As discussed previously, the SuM was found to have direct fastigial inputs by Fujita et al., suggesting a possible disynaptic route to the hippocampus – although, as noted above, no evidence was found in Fujita et al. for a disynaptic pathway to the hippocampus (Fujita et al., 2020). However, the medial septum and diagonal band were found to have likely disynaptic inputs from the FN by Fujita et al., suggesting a possible trisynaptic pathway from the DCN to the hippocampus. Additional areas labeled by rabies due to putative monosynaptic inputs to the hippocampus include the raphe, striatum, and locus coeruleus (Watson et al., 2019), underscoring the possibility of multiple potential pathways from the cerebellum to the hippocampus. However, it remains to be shown that any of these areas do indeed represent such connectivity.

Interestingly, already at 48 h (suggestive of disynaptic input to the hippocampus), some cells were labeled in the DCN (Figure 1D). However, these were extremely limited, and no labelled cells were seen in the cerebellar cortex at this time point. In one animal, three cells were also present in the vestibular nucleus. Areas with heavier labeling at 48hrs included the periaqueductal grey (PAG), the laterodorsal tegmental nucleus (LDTg), and other pontine nuclei (Watson et al., 2019). Note that both the PAG and LDTg receive direct inputs from the DCN (Figures 1A–C) (Teune et al., 2000; Fujita et al., 2020; Kang et al., 2021; Streng et al., 2021; Novello et al., 2022). Fairly strong labeling was also present in the nucleus incertus at this time point (Watson et al., 2019), which may reflect connectivity via (CA1) somatostatin interneurons (Szõnyi et al., 2019), and represents another potential route from cerebellum to hippocampus.

By 58 h after rabies injection into the dorsal dentate gyrus of the hippocampus, rabies labeling was present in bilateral deep cerebellar and vestibular nuclei – including strong labeling in the dentate and fastigial DCN (Figure 1E). Labeling in the FN was concentrated in the caudal portion of the nucleus (Watson et al., 2019), fitting with FN neurons with projections to areas like the thalamus, superior colliculus, PAG, LDTg, zona incerta, and SuM (Figures 1B, C) (Fujita et al., 2020; Streng et al., 2021). Interestingly, sparse expression was also already visible within the cerebellar cortex itself at the 58 h timepoint, and this was much stronger at 66 h (Figure 1D; Watson et al., 2019). At 66 h post injection, the strongest labeling within the cerebellar cortex was observed in the central cerebellum and the ipsilateral paraflocculus, though some labeling was also present in the posterior cerebellum (particularly in lobule IX), and in the anterior cerebellum (particularly in the simplex) (Figure 1F) (Watson et al., 2019). Within the central cerebellum, strong labeling was seen in lobule VI and Crus I, with less strong labeling in lobule VII (Figure 1F). Interestingly, across the cerebellum, rabies positive Purkinje cells were clustered and zebrin-positive (Watson et al., 2019).

Together, the results from Watson et al.’s rabies tracing work suggests distributed, multi-synaptic, input from the cerebellum – with bilateral vestibular, dentate, and fastigial DCN labeled – but also restricted input from the cerebellum, seen as clusters of Purkinje cells in zebrin-positive zones in specific lobules (Figures 1D–F). This work establishes that there are likely multiple pathways by which the cerebellum can influence the hippocampus. Additional pathways may become apparent with additional research. For example, new experiments with starter sites in the ventral CA1 may indicate additional cerebellar regions that connect to the hippocampus, as well as new potential intermediary structures. It should also be noted that this work emphasizes another important point – although we do not yet know (all) the pathways by which the cerebellum influences the hippocampus, and how these relate to specific functions, there *are* specific pathways. At the time points examined, select subsets of cells in specific areas were labeled – it is not merely that at enough synapses, any two regions or neurons in the brain may be connected. Just as there are specific, multi-synaptic, routes by which the cerebellum influences the neocortex, there are specific routes by which the cerebellum influences the allocortex, including the routes contained within the labeled cells in Watson et al. (2019).

We have focused thus far exclusively on the cerebellum to the hippocampus as much less is known about the hippocampus to the cerebellum. Based on how the neocortex influences the cerebellum, one can speculate that it involves the pontine nuclei and mossy fiber inputs (Mitra and Snider, 1975). However, this may possibly be via neocortex intermediate(s) (Schmahmann, 1996; Kelly and Strick, 2003; Takehara-Nishiuchi, 2018). New strategies which allow researchers to better directly connect all the dots (e.g., modified rabies virus coupled with retrograde AAV vectors) will be helpful as we continue to slowly unravel the mystery of which pathways underlie the bidirectionally-connected hippobellum.

## Acute demonstrations of functional connectivity

Early electrical stimulation work supported the bidirectional nature of the hippobellum: work done in the seventies found that electrical stimulation of the cerebellum produced short latency responses in the hippocampus (< 20 ms) (Heath and Harper, 1974; Snider and Maiti, 1976; Heath et al., 1978; Newman and Reza, 1979), and stimulation of the hippocampus evoked responses in the cerebellum (latencies between ∼8-30 ms) (Sager et al., 1970; Newman and Reza, 1979). Electrical stimulation has a number of associated caveats, but work examining epileptiform discharges, discussed more later in this review, further supports the idea that the hippocampus can have acute impacts on the cerebellum (Mitra and Snider, 1975; Heath et al., 1980; Krook-Magnuson et al., 2014). Many of the caveats of electrical stimulation can also be avoided using optogenetic approaches, and multiple studies have examined the impact of acute cerebellar optogenetic manipulation on the hippocampus.

**FIGURE 2:**
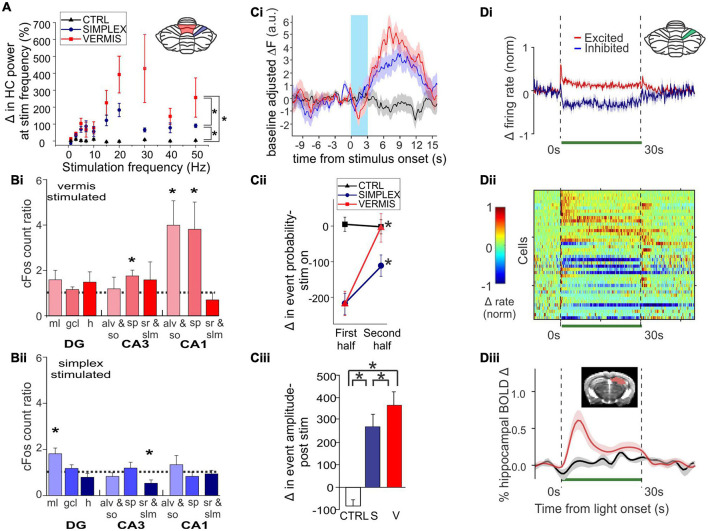
Acute cerebellar modulation alters activity in the hippocampus. **(A)** Activation of Purkinje cells with channelrhodopsin (ChR) in the vermis (red symbols) or simplex (blue symbols) increases hippocampal LFP power at the stimulation frequency, across many frequencies. Inset: Schematic showing lobule IV/V vermis (red shading) and simplex (blue shading), which are targeted in panels **(A-C)**. Black symbols: Opsin-negative controls. **(B)** Activation of Purkinje cells in the vermis **(Bi)** or simplex **(Bii)** alters cFOS expression in multiple hippocampal subregions and layers, suggesting region-specific recruitment of excitatory and inhibitory cell populations. **(C)** Cerebellar stimulation also alters calcium activity in CA1, measured *in vivo* using miniscopes. **(Ci)** Calcium fluorescence prior to, during (blue shaded region), and after cerebellar stimulation, showing a decrease during stimulation and an increase after stimulation. Red trace: Vermis stimulation. Blue: Simplex stimulation. Black: Opsin-negative controls. **(Cii)** Analysis of deconvolved calcium data shows a transient decrease in event probability during light delivery, especially in the first half of the stimulation. No such effect was seen in opsin-negative controls. **(Ciii)** A large increase in event amplitude was seen after light delivery offset. **(D)** Inhibition of Purkinje cells with Archaerhodopsin (Arch) near the simplex (green shading in inset) alters activity of cells in the hippocampus, shown at the level of single units and via a population Blood-Oxygen Level Dependent (BOLD) signal. **(Di)** Summarized data from (Dii), showing that some hippocampal cells are excited (red trace), while others are inhibited (blue trace), by light delivery to the cerebellum (green bar). **(Dii)** Single unit recordings during inhibition of Purkinje cells. Cool colors represent decreased firing rate; warm colors represent increased firing rates. Note also the bursts of activity seen in some units at light offset. **(Diii)** Cerebellar manipulation results in an increased BOLD signal in the hippocampus (red trace: Arch; black: Opsin-negative control). Inset: Mask used for area of BOLD measurement. Together these data show that acute cerebellar manipulation impacts activity in the hippocampus, and likely influences populations of both principal cells and interneurons. Panels **(A-C)** were reproduced with modification from Zeidler et al. (2020) panels **(A-C)** under a CC-BY 4.0 license. Panel **(D)** was modified slightly for arrangement from Choe et al. (2018) with permission from Elsevier.

Zeidler et al. (2020) used optogenetics to acutely activate Purkinje cells in either the simplex or the vermis (lobule IV/V) and examined a range of impacts on the hippocampus. Most closely fitting to earlier electrical stimulation studies, Zeidler et al. found that brief light activation of Purkinje cells (in either cerebellar area) produced a hippocampal local field (LFP) response (Figure 2A). The latency from the start of light onset (10 ms pulse width) to the peak of the initial response in the hippocampus was ∼13-16 ms. A range of stimulation frequencies were examined, with the strongest hippocampal responses being evoked at frequencies above 15Hz (Figure 2A). Optogenetic activation also increased cFos expression in the hippocampus, but only in particular regions (Figure 2B). Specifically, significant increases were seen in the dentate gyrus molecular layer (home to inhibitory interneurons) following simplex stimulation and in the stratum pyramidale (a principal cell layer) of CA3 and CA1 and the alveus/oriens (containing generally inhibitory interneurons) in CA1 following vermal stimulation. This data shows that cerebellar activation does not simply produce a generalized activation of the entire hippocampus but instead engages both excitatory and inhibitory elements in specific subregions. Zeidler et al. also monitored CA1 activity using miniscopes and calcium imaging. Overall, simplex or vermal stimulation led to an acute decrease in event probability during the time of light delivery followed by a very large rebound in event amplitude (Figure 2C).

Acute effects of cerebellar optogenetic manipulation on the hippocampus were also examined in work conducted by Choe et al. (2018). Fitting with data discussed above, single unit recordings from the hippocampus showed both inhibitory and (bursty) excitatory responses to acute inhibition of the cerebellar cortex near the simplex (Figures 2Di, Dii). Nearly 70% of dorsal hippocampal neurons showed some form of modulation. The majority of those were inhibited, but 18% showed some form of excitation, often bursting at onset and/or offset (Figures 2Di, Dii). Choe et al. additionally took a whole brain view of areas modulated by this cerebellar manipulation by doing fMRI concurrent with optogenetics (termed ofMRI). Several pieces of key information come from this work. First, the hippocampus is activated by their acute cerebellar manipulation, as seen by increases in the BOLD signal (Figure 2Diii). Second, this increase happens with very short (for a BOLD response) delay from the start of cerebellar manipulation (fitting with the single cell, calcium imaging, and LFP data already described). Third, while the hippocampus was one of several brain areas to show a BOLD response, BOLD responses occurred in only select areas (i.e., it was not a brain-wide response). The contralateral hippocampus had 21% of voxels activated. For comparison, the motor cortex showed 22% activation. The reticular formation, thalamus, anterior cingulate and retrosplenial cortices were among the additional areas showing increases (70, 46, 25, and 23% respectively). In contrast, basal ganglia showed only 2%, somatosensory cortex 5%, and rhinal and insular showed 0% (Choe et al., 2018). This highlights that effects of the cerebellum on hippocampal activity are specific – cerebellar manipulation does not create BOLD changes in the entire brain but rather in a subset of regions that includes the hippocampus.

## Functional collaborations – Coordinated oscillations

While the above studies demonstrate connectivity of the hippocampus and the cerebellum, they provide limited insight into the function of such connectivity – why do these two seemingly disparate brain regions talk to one another? What information are they providing to each other? What functions in each brain region does this communication help support? While many more questions than answers remain, research is providing important insight into the potential functions and conditions under which the cerebellum and the hippocampus collaborate. We discuss first their coordinated oscillatory activities. Context-dependent coherent oscillations are detected in brain regions collaborating on specific tasks, and such oscillations are thought to help with information transfer between regions (Fries, 2005; Jones and Wilson, 2005; Sirota et al., 2008; Hyman et al., 2010; Colgin, 2011; Spellman et al., 2015). Coherent oscillations have been noted between the hippocampus and the cerebellum, by several labs and in several contexts.

**FIGURE 3:**
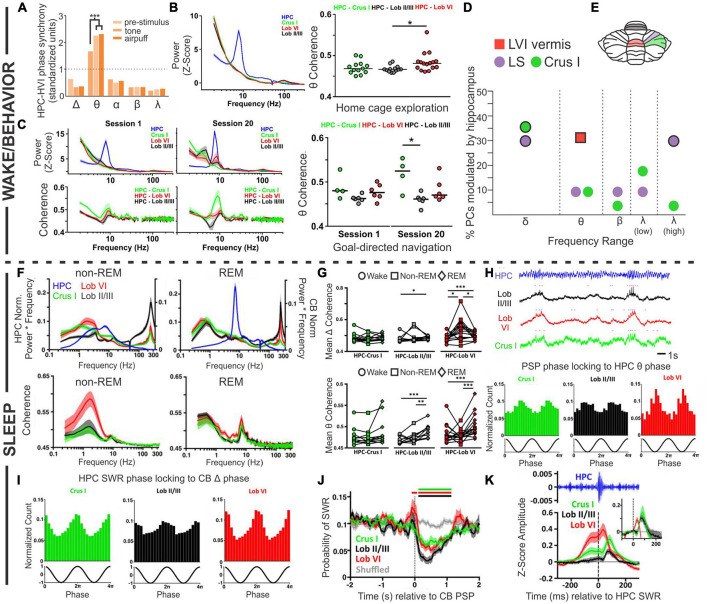
Coordinated oscillations. Coordination between the cerebellum and the hippocampus during wake or active behaviors **(A–D)** or during sleep states **(F–K)**. **(A)** During trace eyeblink conditioning in rabbits, the hippocampal (HPC) and cerebellar hemisphere lobule VI (HVI) theta (θ) oscillations show phase synchrony. This coherence is not found for other frequencies and is increased by conditioned (tone) or unconditioned (airpuff) stimulus presentation. **(B)** Left: During home cage exploration, the hippocampus (blue trace) but not the cerebellum (green: Crus 1; black: lobule II/II; red: lobule VI) shows high theta power. Right: The two structures *do* however, display *coherence* in theta oscillations during home cage exploration, with the highest levels of theta coherence with the hippocampus seen in lobule VI. **(C)** Power in each brain region (top) and cerebellar coherence with hippocampal oscillations (bottom) early during training (Session 1, left) and late during training (Session 20, middle), on a linear track with reward locations. Interestingly, by Session 20, there is a slight peak in lobule VI power in the theta range (top middle, red peak) that is not present on the first day of training (top left). Coherence between the structures in Session 1 is similar to what is seen during home cage exploration (compare to panel B), but on Session 20, when the animals are fully trained, there is increased coherence between the hippocampus and Crus I (middle bottom, green peak). Right: Summary theta coherence data across animals, by session and cerebellar region. **(D)** Cerebellar single units were phase locked to different hippocampal frequencies with regional differences: Purkinje cells (PCs) in simplex lobule (LS; lilac circles) phase locked with both delta (peaking at 1Hz) and high gamma (peaking at 60 Hz), while Crus I PCs (green circles) were modulated only by hippocampal delta (peaking at approximately 1.5 Hz and 2.5 Hz). PCs in lobule VI (LVI; red square) were modulated by hippocampal theta (6-12 Hz) specifically during active locomotion. Frequencies at which modulation was found to be significant in each region are outlined in black. **(E)** Rodent cerebellar flatmap showing the regions of the cerebellum described throughout the figure. Color coding consistent with other figure panels. **(F–K)** During sleep, the cerebellum and hippocampus show coordinated activity across sleep stages. **(F)** Power (top) and coherence (bottom) of oscillations in and between the cerebellum and the hippocampus during non-REM and REM sleep. Note that the y-axis labels (left and right) apply to both top panels. The hippocampus has high theta during REM sleep, while cerebellar oscillations are dominated by activity in the delta frequency range, in addition to very high frequency (∼250 Hz) activity, in both sleep stages. During non-REM sleep coherence between the structures is most prominent in the delta frequency (bottom left), while during REM sleep all three CB regions show a peak of coherence in the theta range (bottom right). **(G)** A summary of coherence in delta (top) and theta (bottom) ranges, comparing wake (circles), non-REM (squares), and REM (diamond) states. (**H**, top) All cerebellar regions examined display phasic sharp potentials (PSPs) (pink dots in traces represent PSP occurrences). (**H**, bottom) PSPs in all regions are phase locked to hippocampal theta. **(I)** Sharp wave ripples (SWRs) in the hippocampus during non-REM sleep phase lock to cerebellar delta phase. **(J)** Prior to cerebellar PSP there is an increase probability of hippocampal SWRs, and post cerebellar PSP there is a decreased probability of hippocampal SWRs. **(K)** Hippocampal SWRs are associated with evoked potentials in the cerebellum. Taken together, these studies each support the bidirectional coordination of the hippocampus and the cerebellum across tasks, brain states, and time scales. The graph in **(A)** has been recreated from data in Wikgren et al. (2010) with permission from Elsevier. The graph in **(D)** depicts a combination of data from McAfee et al. (2019) (LS and Crus I; circles) and (Watson et al., 2019) (Lob VI; square). Other panels in this figure have been reproduced with slight adaptations from Watson et al. (2019) **(B,C)** and (Torres-Herraez et al., 2022) **(F–K)**, both under a CC-BY 4.0 license.

One example of task-specific coherent cerebellar-hippocampal oscillations comes from trace eyeblink conditioning, with work often done in rabbits. Note that rabbits can show strong hippocampal theta during trace eyeblink conditioning, even though not running (Hoffmann et al., 2015). Trace eyeblink conditioning is a version of eyeblink conditioning which, in addition to being cerebellar dependent, is also hippocampal dependent – as discussed more in later sections and reviewed in Takehara-Nishiuchi (2018). Wikgren et al. (2010) recorded from the hippocampus and lobule VI in the cerebellum, and found that the power of theta (or more specifically, theta to delta ratio) in the hippocampus and cerebellum corresponded to one another; high theta in the cerebellum was noted when the hippocampus was in a theta state. Moreover, they observed coherence in cerebellar and hippocampal theta oscillations (i.e., phase synchrony) during trace eyeblink conditioning, especially during high hippocampal theta states (Figure 3A). Coherence increased in response to the conditioned or unconditioned stimulus (even in already high theta states) but decreased over the course of training. Notably, they did not observe significant coherence for any of the other frequency bands examined (delta, alpha, beta, gamma) (Figure 3A). Hoffmann and Berry (2009) likewise found that when training occurred during theta states – in which they saw covariant theta time locked to the sensory stimuli in the cerebellar cortex, cerebellar interposed nucleus, and hippocampus – there was an enhancement of learning. A relationship between hippocampal theta state and eyeblink conditioning has been observed in several studies (Berry and Thompson, 1979; Seager et al., 2002; Nokia et al., 2008).

Another example of coordination between the hippocampus and the cerebellum comes from experiments in which cerebellar (Crus I, lobule II/III, or lobule VI) and hippocampal theta were examined during spontaneous homecage locomotion in mice (Watson et al., 2019). In contrast to findings during eyeblink conditioning, under these conditions Watson et al. found a strong theta oscillation only in the hippocampus and not in the cerebellum, at any recorded cerebellar location (Figure 3B). Coherence analysis, however, examining the relationship between oscillations in the hippocampus and cerebellum, did show a strong peak at theta (Figure 3B). Therefore, cerebellar-hippocampal theta coherence does not require a high theta amplitude *per se*. Cerebellar-hippocampal theta coherence occurred for all recorded cerebellar locations. It was overall the strongest in lobule VI, but there was considerable overlap between distributions at each location (Figure 3B). Note that lobule VI is one of the areas showing the strongest rabies labeling 66 h after injection into the hippocampus (Watson et al., 2019; Figure 1). Watson et al. also examined firing of single cerebellar neurons under head-fixed conditions and found that 31% of putative Purkinje neurons in lobule VI showed significant phase locking to hippocampal theta (Figure 3).

Compared to home cage locomotion, a slightly different picture emerged when Watson et al. examined the progression of coherence over several days of training on a goal-directed task (i.e., a linear track with reward zones on either end) (Watson et al., 2019). At the start of training, findings were overall very similar to home cage locomotion – a clear theta band was present in the hippocampus, but lacking from the cerebellum, and there was coherence between the hippocampus and the cerebellum in the theta range (Figure 3C). Which cerebellar region in particular had the greatest coherence showed some variability between the two conditions (home cage locomotion versus goal-direct linear track), suggesting some possible variability in the data or task-dependency. The greatest difference between the data sets, however, appeared once the animals were well-trained on the task. At the end of training (session 20), a theta peak *was* present in the cerebellum, chiefly in lobule VI (Figure 3C). Combined with data from eyeblink conditioning recordings described above, this suggests a possible influence of learning or task-dependency on the appearance or lack of cerebellar theta.

At all stages of training examined by Watson et al., cerebellar theta power in all recorded locations was positively correlated with mouse running speed (Watson et al., 2019). Cerebellar-hippocampal theta coherence, however, was not correlated with running speed early in training. Late in training, a region-specific profile emerged: cerebellar-hippocampal theta coherence was positively correlated with running speed in lobule VI, showed no correlation for Crus I, and interestingly, was actually negatively correlated with speed for lobule II/III. Despite not showing a correlation with running speed, Crus I theta coherence with the hippocampus was very strong at the well-trained stage in the task (Figure 3C). This suggests that not only do different cerebellar regions likely interact in different ways with the hippocampus but also that these interactions are dynamic and change during task learning. Note that Crus I also showed strong rabies labeling 66hrs after injection into the hippocampus (Figure 1F; Watson et al., 2019).

Rondi-Reig and colleagues, in work led by Torres-Herraez, additionally examined cerebellar oscillations and their relationship to hippocampal oscillations during sleep using many of the same animals as in Watson et al. (2019), Torres-Herraez et al. (2022). While the hippocampus displays a high theta to delta ratio during REM sleep (and not during non-REM sleep; almost by definition) (Montgomery et al., 2008; Mizuseki and Miyawaki, 2017; Girardeau and Lopes-Dos-Santos, 2021), Torres-Herraez et al. (2022) found high cerebellar delta (< 4 Hz) in either sleep state, although delta power was often higher during non-REM (especially in lobule VI). Very high frequency oscillations (∼250 Hz), across all cerebellar regions (Crus I, lobule VI, lobule II/III) and all behavioral states examined, were also noted, though they were greatest during sleep states (with non-REM greater than REM) and in lobule II/III (Figure 3F). Hippocampal-cerebellar delta coherence profiles during REM sleep largely mirrored those seen in wake (described above), but there was an increase delta coherence with the hippocampus during non-REM sleep, especially between the hippocampus and lobule VI (Figures 3F, G). Theta coherence, in contrast to delta coherence, showed little region to region variability across states (also somewhat in contrast to the locomotor analysis above), but did show state-dependence, with REM sleep typically showing the highest levels of cerebellar-hippocampal theta coherence (Figures 3F, G).

Torres-Herraez et al. (2022) also describe cerebellar phasic sharp potentials (PSPs) (Figure 3H). These high amplitude (∼2.3 standard deviations from baseline), brief (∼130 ms duration) events often occurred simultaneously across cerebellar brain regions. Cerebellar PSPs occurred in clusters during REM sleep (interevent interval < 1s) and were less frequent and more distributed (less clustery) during non-REM sleep. Cerebellar PSPs showed strong phase locking to the peak of cerebellar REM delta oscillations, and less strong but still prominent phase locking to the trough of hippocampal REM theta oscillations (Figure 3H). This illustrates a coordination of both oscillations (e.g., delta and theta) and short events (i.e., cerebellar PSPs to hippocampal theta) across brain regions.

Conversely, hippocampal sharp-wave ripples (in non-REM sleep) were phase locked to cerebellar delta waves (Figure 3I; Torres-Herraez et al., 2022), indicating that fine-grained coordination between the cerebellum and the hippocampus occurs in both directions. Moreover, when cerebellar PSPs occurred during non-REM sleep, hippocampal sharp-wave ripples were suppressed ∼90 ms later (for about 1 s), regardless of where the cerebellar PSP was recorded (Figure 3J). This suggests a possible cerebellar influence on the hippocampus (or a reflection in both structures of some other unifying phenomenon) during sleep. Collectively, these data also fit with, and extend in important new directions, recent work suggesting cerebellar influences on sleep spindles (Xu et al., 2021) and sleep-wake transitions (Zhang et al., 2020).

When hippocampal sharp-wave ripples did occur, short latency (∼10 ms for lobule VI; ∼30 ms for the other locations) LFP responses were noted in all recorded cerebellar locations (Torres-Herraez et al., 2022), suggesting hippocampal influence on the cerebellum in sleep and again highlighting the bidirectional nature of hippobellum interactions (Figure 3K). Additionally, an increase in the probability of hippocampal sharp-wave ripples was noted 100 ms prior to cerebellar PSPs (Figure 3J), potentially indicating that hippocampal sharp-wave ripples can promote cerebellar PSPs, somewhat akin to ripple-spindle coupling with the cerebral cortex (Siapas and Wilson, 1998).

While we have discussed the cerebellum as an area sometimes showing coherence with hippocampal oscillations and examples of both structures appearing to influence oscillations in the other region, it is important to note that a different line of thinking considers the cerebellum at the top, as a supervisor of oscillations across brain regions (Popa et al., 2013; Lindeman et al., 2021; McAfee et al., 2022). This need not be exclusive of the cerebellum having other direct impacts on the hippocampus, nor would it exclude the possibility for the hippocampus to influence the cerebellum. The concept of the cerebellum as an oscillation coordinator applies to other regions besides just the hippocampus, but we will focus on it here in the context of the hippobellum. The cerebellum may be important in the coordination of activity in the hippocampus with activity in other brain areas, including the medial prefrontal cortex (mPFC). For example, McAfee et al. found that cerebellar Purkinje cells’ firing could represent not only the phase of oscillations in the hippocampus (Figure 3D) (and the mPFC), but also the phase *differences* between the hippocampus and the mPFC (McAfee et al., 2019). Interestingly, some regional differences in these representations were noted across the cerebellum. Specifically, simplex neurons showed significant representation of mPFC and CA1 phases for delta and gamma frequencies, but in Crus I this only occurred for delta frequencies. However, a somewhat different profile was observed for representation of phase *differences* between mPFC and CA1. Theta was the most heavily represented frequency range in the simplex, but delta, beta, and low and high gamma were also represented. Crus I also captured most of the spectrum (including delta, theta, beta, and low gamma) (McAfee et al., 2019). It is interesting that within the simplex, differences in *theta* phase between the mPFC and CA1 were the most heavily represented, as these recordings were done in head-fixed (presumed awake) animals at rest – and therefore likely during relatively low theta states. It would be interesting to see how the power of theta in either the mPFC or hippocampus would impact the representation of phase differences within the cerebellum, or as seen for other measures discussed above, how it might change across tasks, brain states, and learning paradigms.

Taken together, these studies illustrate that the cerebellum and the hippocampus show coherent oscillatory activity across different tasks and brain states, and this coordinated activity may be important for task performance. The cerebellum may additionally monitor (and potentially influence) phase differences between the hippocampus and other structures, such as the mPFC, providing another avenue for hippobellum interactions.

## When timing is of the essence

Appropriate timing is critical for classical cerebellar functions (Ivry et al., 2002; O’Reilly et al., 2008; Cheron et al., 2016), and in some circumstances, the hippocampus may provide critical support to the cerebellum for such purposes. Much the way hippocampal “place cells” encode location and spatial trajectories (Hartley et al., 2014; Chao et al., 2020a), hippocampal “time cells” are believed to encode temporal components (reviewed in Eichenbaum, 2014; Banquet et al., 2021). This hippocampal ability may come into play in trace eyeblink conditioning, and other forms of time-predictive motor responses that also involve the cerebellum.

**FIGURE 4:**
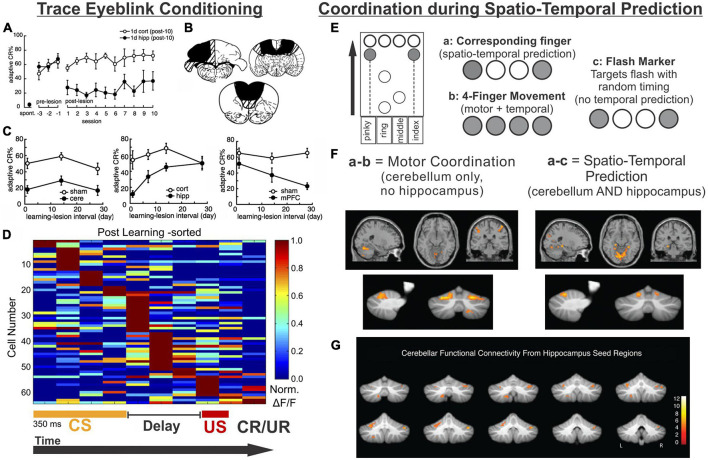
The hippocampus may help the cerebellum parse time. **(A–D)** Trace eyeblink conditioning is a task that requires coordination between the cerebellum (cere) and the hippocampus (hipp) for successful task performance. **(A)** Lesions to the hippocampus made one day after learning a conditioned response impaired performance (closed black circles) compared with animals that received cortical lesions (open circles). **(B)** Example lesions of the cerebellum, prefrontal cortex, or hippocampus for the graphs in **(C)**. **(C)** (left) Lesions to the cerebellum permanently disrupt performance, while lesions to the hippocampus prior to (not shown) or shortly after learning (middle) are detrimental. Lesions to the PFC (right) are more detrimental later after learning. **(D)** Calcium activity in hippocampal cells was imaged over the course of learning a trace eyeblink conditioning paradigm. Shown are activity patterns once the task is learned. Hippocampal cells tile the temporal space across both stimuli and the stimulus-free trace period (a function of “time cells”). These data suggest the hippocampus may be critical for trace eyeblink conditioning because it is providing temporal information to the cerebellum about when stimuli are occurring and when a response is necessary **(E–G)**. The cerebellum and hippocampus also coordinate during finger-tapping task with a spatio-temporal prediction component **(E)**. Subjects tapped 1 or 2 keys when the corresponding circles scrolled onto the targets at the top of the screen (condition a) or when the targets flashed (condition c). In the 4-finger condition (b), the participants tapped all 4 keys when any marker reached the target. **(F)** Subtracting the activity of the 4-finger movement condition from the corresponding finger condition allowed for extraction of regions associated with motor coordination (left) (and subtraction of temporal processing activity patterns). Conversely, subtracting the activity of the flash marker condition from the corresponding finger condition allowed for extraction of regions involved in temporal-prediction (right). The hippocampus and the cerebellum were coactivated when temporal prediction was necessary. **(G)** When a seed region in the left hippocampus was chosen for a functional connectivity analysis, lobule VI, lobule VIIIb, and R Crus I of the cerebellum were all functionally connected with the hippocampus. Data and schematics in **(A-C)** reproduced and modified for arrangement in this figure from Takehara et al. (2003) (Copyright 2003 Society for Neuroscience). Data in panel **(D)** reproduced from Modi et al. (2014) with minor labeling modifications under a CC-BY 3.0 license. Schematics in E based on (Onuki et al., 2015). Images in panels **(F,G)** were reproduced from parts of figures in Onuki et al. (2015) with permission from Oxford University Press. CS, conditioned stimulus; US, unconditioned stimulus; CR, conditioned response; UR, unconditioned response.

As recently reviewed by Takehara-Nishiuchi (2018), there are several variations of eyeblink conditioning, and all of them require an intact, functioning cerebellum (McCormick and Thompson, 1984; Woodruff-Pak et al., 1985; Weible et al., 2000; Takehara et al., 2003). Classical conditioning typically has an air puff (unconditioned stimulus [US]) delivered to the eyelid, which then results in a reflexive blink. In following trials, the air puff is preceded by a visual or auditory cue (conditioned stimulus [CS]). The US co-terminates with the CS in the standard version of this task, and after enough pairings, the conditioned stimulus alone will result in a blink (conditioned response [CR]). *Trace* eyeblink conditioning is a task variation in which a stimulus-free gap (i.e., the trace period) is introduced between the conditioned and unconditioned stimulus, making a successful association of the CS and US take more trials (Sears and Steinmetz, 1990; Clark and Squire, 1998; Tseng et al., 2004; Walker and Steinmetz, 2008). Interestingly, the introduction of this delay, which generally ranges between 250 ms and 1,000 ms (depending on species) (Woodruff-Pak and Disterhoft, 2008), converts the task into one which relies on the hippocampus to learn: impaired hippocampal function impairs the acquisition of trace eyeblink conditioning (Solomon et al., 1986; Moyer et al., 1990; Kim et al., 1995; Clark and Squire, 1998; Weiss et al., 1999; Takehara et al., 2002, 2003; Weiss and Disterhoft, 2015). Several studies have shown that when the hippocampus is lesioned shortly after learning (e.g., a day or a week), performance is also impaired (Figures 4A–C; Kim et al., 1995; Takehara et al., 2002, 2003). In contrast, performance further after initial learning – as the memory becomes remote – relies more on neocortical structures like the mPFC (Figure 4C; Takehara et al., 2003; Takehara-Nishiuchi, 2018).

While hippocampal lesions only disrupt the learning of certain forms of eyeblink conditioning (including trace), hippocampal activity also reflects the conditioned stimulus in classical eyeblink conditioning (Berger et al., 1976; Patterson et al., 1979; Clark et al., 1984; Sears and Steinmetz, 1990; Ryou et al., 1998). This suggests a broader engagement of the hippocampus in eyeblink conditioning, even if obvious deficits are not always noted. For example, Clark et al. found that CA1 neurons displayed an increase in firing around the US early during classical conditioning, but with conditioning, this shifted to be during the CS, and this shift in firing paralleled the shift of the behavioral response (Clark et al., 1984). Interestingly, while hippocampal activity is not required for acquisition of classical eyeblink conditioning, such hippocampal activity during classical eyeblink conditioning does require the cerebellum. Specifically, lesion of the cerebellar interposed or dentate nucleus had a strong effect on the hippocampal responses to the CS, essentially abolishing them (Clark et al., 1984; but see also Sears and Steinmetz, 1990). This again highlights the *bidirectional* functional connectivity between the hippocampus and the cerebellum and supports a cognitive collaboration between the structures even during classical eyeblink conditioning.

Why is the hippocampus needed for trace eyeblink conditioning? One theory is the hippocampus may bridge the temporal gap of the trace interval (Hoffmann et al., 2015). More specifically, the hippocampus is likely providing key temporal information (i.e., specifically *when* the airpuff (US) will happen after the CS). As noted above, the hippocampus is able to encode timing of events via “time cells” (Manns et al., 2007; MacDonald et al., 2011), and this may be its key contribution to trace eyeblink conditioning. In seminal work by MacDonald and colleagues, rats had to pair an object with one of two odors after a 10 s delay, and cells in CA1 preferentially represented particular time windows in the delay period (MacDonald et al., 2011). The entire time window of the delay was represented fully by tiling of these “time cells” in the hippocampus, allowing for a temporal coding of the object presentation and the odor presentation.

A number of studies examining hippocampal activity during trace eyeblink conditioning support the idea that the hippocampus is providing temporal support to the cerebellum during the task (Solomon et al., 1986; Weiss et al., 1996; McEchron and Disterhoft, 1999; Weible et al., 2006; Green and Arenos, 2007; Modi et al., 2014). One notable study by Modi and colleagues used two-photon calcium imaging to examine hippocampal activity during the training of trace eyeblink conditioning (Modi et al., 2014). Some cells were highly active during the CS, some were highly active during the delay, and some were highly active during the US (Modi et al., 2014). Importantly, during the trace period, populations of neurons showed time-specific firing (i.e., were “time cells”), and together represented the entire trace period (Figure 4D). This is very similar to the tiling of time noted in the object-odor pairing task described above (MacDonald et al., 2011). Two more recent studies have provided information also about the activity and role of hippocampal inhibitory interneurons during trace eyeblink conditioning – hippocampal interneurons actively increased their firing rates during the CS and the US (Zhang et al., 2021; Li et al., 2022), and inhibition of broad (Zhang et al., 2021) or specific (Li et al., 2022) interneuron populations disrupted task acquisition. Taken together, these studies suggest that hippocampal time cells may bridge the temporal gap between the CS and US (during acquisition) and illustrate that the importance of inhibition to proper hippocampal functioning should not be ignored. The need for the hippocampus during acquisition of trace eyeblink conditioning suggests that this temporal processing across the trace gap cannot be achieved by the cerebellum without the hippocampus.

The role of the hippocampus for associating two (relatively) distant-in-time stimuli is supported in other types of associative learning as well, including fear conditioning, which is also impaired by hippocampal damage (Marchand et al., 2004). It has also been shown that the hippocampus can play a more general role in forming associations, including environmental context, during eyeblink conditioning (Penick and Solomom, 1991; Takehara-Nishiuchi, 2018). More broadly, the hippocampus is associated with episodic memories (recently reviewed in Eichenbaum, 2017; Chao et al., 2020a), and it has been suggested that hippocampal-supported conscious awareness of the association between the US and CS is key to trace eyeblink conditioning (reviewed in Clark et al., 2002). This may relate to increased learning during attentional theta states (Berry and Thompson, 1979; Seager et al., 2002; Nokia et al., 2008). Ultimately, the hippocampus likely encodes several aspects of eyeblink conditioning and may be supporting the cerebellum in its execution of the eyeblink response through multiple mechanisms.

In trace eyeblink conditioning, it appears that the hippocampus provides, among other things, critical task-related temporal support to the cerebellum. The hippocampus providing predictive timing support to the cerebellum occurs in other circumstances as well, as illustrated by an fMRI study by Van der Werf and colleagues in which participants performed several finger-tapping tasks (Onuki et al., 2015). The finger-tapping tasks varied in task demands, allowing for contrasts to examine activation associated with specific task components (Figure 4E). Three task variations are particularly relevant to this discussion. In one (‘flash marker’), participants pressed buttons when targets flashed. This version had a finger-selection/spatial component (as the location of the flash indicated which fingers to press), but no timing-prediction component (as the inter-flash intervals were varied and could not be predicted). In another version of the task (‘4-finger movement’), markers scrolled across the screen, and subjects pressed all four fingers whenever any of the markers reached the target. It therefore had a timing-prediction component (as one can predict when the markers will reach the targets based on scrolling speed), but no finger-selection component. The third version (‘corresponding finger’) required pressing the appropriate keys (one or two fingers) when the associated markers reached their targets, and thus had both a finger-coordination and a timing-prediction component.

When the ‘4-finger movement’ condition was subtracted from the ‘corresponding finger condition’, the cerebellum but not the hippocampus showed activation. This supports that the cerebellum is relevant for the finger selection task component (i.e., motor coordination). Note that both of these conditions have the temporal-prediction component, and thus activation associated with that aspect of the task would be subtracted out. In contrast, subtracting activation during the “flash marker” condition from the “corresponding finger” condition removed activation associated with the finger selection component of the task and highlighted areas selectively activated by the temporal prediction component. This contrast revealed coactivation of the cerebellum (in particular lobule VI) and the hippocampus (Figure 4F; Onuki et al., 2015). Functional connectivity analysis using the left hippocampus as a seed region further supported task-related hippocampal connectivity with the cerebellum, including in lobule VI and Crus I (Figure 4G; Onuki et al., 2015). Therefore, in both trace eyeblink conditioning and a finger tapping task, the hippocampus is engaged and likely providing key *when* information for the cerebellum.

## Time is not the final frontier: Hippobellum in spatial processing

The hippocampus certainly plays an important role in temporal processing, but its role as a major hub for *spatial* processing has also been well studied and supported (O’Keefe and Dostrovsky, 1971; McNaughton et al., 1983) (reviewed in Broadbent et al., 2004; Witter et al., 2014; Nyberg et al., 2022). The cerebellum may also play critical roles in spatial processing. It receives multimodal information from cortical, subcortical, and brainstem regions and is an integrator of both motor and non-motor systems (Azizi and Woodward, 1990; Huang et al., 2013; Ishikawa et al., 2015; Popa et al., 2019), a feature that would be helpful in navigation of one’s surroundings. Indeed, humans with cerebellar damage, including those with Cerebellar Cognitive Affective Syndrome (CCAS), have trouble with visuo-spatial tasks (Schmahmann and Sherman, 1998). Functional studies in healthy individuals also support the role of the cerebellum in spatial processing. A meta-analysis of fMRI studies found that tasks involving spatial processing activated lobule VI bilaterally, with the strongest activation in the left hemisphere (Stoodley and Schmahmann, 2009), and another more recent fMRI study showed broad cerebellar activation in a spatial mapping task (King et al., 2019). The role of the cerebellum in spatial processing is conserved in other species as well. Hemi-cerebellectomized rodents perform poorly in spatial navigation tasks, though interpretation of results in these studies could be confounded by motor impairments (Petrosini et al., 1996; Mandolesi et al., 2001; Colombel et al., 2004). However, animals with smaller cerebellar lesions and preserved motor capabilities also show deficits in spatial tasks (Joyal et al., 2001; Burguière et al., 2005). Another research group recently showed navigation deficits in a Barnes Maze task when a subpopulation of cells in the cerebellar dentate nucleus was chemogenetically inhibited (Locke et al., 2018). These studies all show that the cerebellum is important for spatial navigation as a whole, but not necessarily how this relates (or doesn’t) to hippocampal functioning and spatial processing in the hippocampus.

A key type of information provided by the cerebellum is an organism’s perception of its movement through space, or self-motion. Self-motion data is compiled from a variety of sources, including but not limited to the vestibular system, and conveyed to Purkinje cells via parallel fibers (PFs) of granule cells and climbing fibers (CFs) of the Inferior Olive (reviewed in Rondi-Reig et al., 2014). Purkinje cells compile this multimodal data to inform an organism’s understanding of how it is moving in relation to its environment. The hippocampus is able to utilize self-motion information during navigation – for example, when healthy rats explore a fully lit arena and form place cells in that arena, turning lights off does not affect hippocampal place cell activity (Quirk et al., 1990) – providing a potential avenue by which the cerebellum might contribute to hippocampal function. Animals will also use environmental cues to determine where they are (recently reviewed in Barry and Burgess, 2014; Danjo, 2020), and here too the cerebellum may play a role, as discussed more below.

**FIGURE 5:**
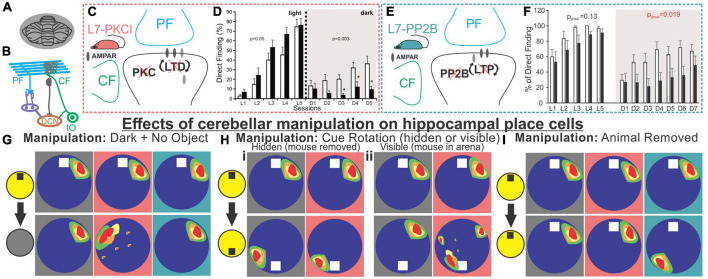
Alteration of Purkinje cell plasticity mechanisms results in impaired spatial processing, with distinct phenotypes. **(A)** L7-PKCI and L7-PP2B mice have genetic mutations which chronically affect cerebellar Purkinje cells across the cerebellum. **(B)** Depiction of climbing fiber (CF) and parallel fiber (PF) inputs to Purkinje cells. **(C)** L7-PKCI mice have chronic impairment of Protein Kinase C (PKC) function in Purkinje cells, disrupting CF-induced long-term depression (LTD) at PF to Purkinje cell synapses. **(D)** In a Morris Water Maze (MWM) task, in which mice are trained to swim to an escape platform, L7-PKCI mice performed comparable to controls in the light (left, L1-L5) but showed marked impairment in the dark (right, D1-D5). **(E)** L7-PP2B mice have impaired long-term potentiation (LTP) due to a loss of PP2B in Purkinje cells. **(F)** L7-PP2B mice also performed poorly on the MWM in the dark (right, D1-D7). **(G–I)** Hippocampal place cells in L7-PKCI mice and L7-PP2B mice were differentially affected by loss of Purkinje cell plasticity in variations of an open field task. Place cells were examined while the mouse explored the arena with an object present and the lights on and then after different manipulations. Schematics of impacts on place cells are shown with a gray background for controls, a salmon-colored background for L7-PKCI mice, and teal for L7-PP2B mice. **(G)** When lights were turned off and the object was removed from the arena, animals were forced to rely on self-motion information. L7-PKCI mice had unstable place fields, whereas L7-PP2B mice showed no deficits compared with controls. Interestingly, if the lights were turned off but the cue/anchor object was still present, L7-PKCI mice had no place cell deficits (not shown), implying spatial processing deficits in L7-PKCI mice specific to reliance on self-motion information. **(Hi)** After initial exploration, the animal was removed from the arena and the cue was rotated. In this condition, both wildtype and L7-PKCI mice place fields rotated according to the new position of the object (i.e., they appear to use the object as an anchor). **(Hii)** When the animal was left in the arena and the cue was visibly rotated – a condition which presents a conflict between object and self-motion cues – wild type animals’ place cells remained stable (i.e., did not rotate with the cue). In contrast, L7-PKCI mice had unstable place fields showing variable levels of rotation (a blurring of place field is shown in the schematic to depict this). This again supports a deficit related to the use of self-motion information in L7-PKCI animals. **(I)** L7-PKCI animals, like controls, show stability of place cells when removed and replaced into a familiar environment, with the lights on and the cue in a stable location. In contrast, L7-PP2B mice occasionally show a spontaneous, coordinated, rotation of place fields, suggesting weaker anchoring to the visible cue. Taken together, experiments outlined in **(G–I)** suggest that L7-PKCI mice may have a deficit in the use of self-motion information, while L7-PP2B mice may have a deficit in the use of anchoring cues. Panel B was modified from Streng and Krook-Magnuson (2020b). Panel **(D)** was reproduced from Rochefort et al. (2011) with permission from AAAS. Panel **(F)** was adapted from Lefort et al. (2019) under a CC-BY 4.0 license. Schematics in panels **(G-I)** were created as representations of data from Rochefort et al. (2011), Lefort et al. (2019).

Rondi-Reig, Rochefort, and others have performed a series of interesting studies providing key insights into how the cerebellum may be influencing the hippocampus using two different mouse lines: 1) L7-PCKI mice, in which Purkinje cells have a dysfunctional protein kinase C (PKC), and thus impaired long-term depression between parallel fibers and Purkinje cells, and 2) L7-PP2B mice, in which Purkinje cells lack protein phosphatase 2B, a protein important for the expression of long term potentiation at parallel fiber synapses (Figure 5; Burguière et al., 2005; Rochefort et al., 2011; Lefort et al., 2019). L7-PKCI animals show normal performance on a cued version of the Morris Water Maze but are impaired on the task in the dark when there is no visual cue (Figure 5; Burguière et al., 2005; Rochefort et al., 2011), suggesting a specific impairment in spatial navigation when only able to use self-motion cues. Paralleling behavioral data, hippocampal place cells in L7-PCKI (like wildtype [WT]) mice are stable in the light, and stable in the dark if there is an anchoring cue (an object) in the arena (Figure 5G; Rochefort et al., 2011). However, without such a cue, L7-PCKI hippocampal place cells are unstable in the dark (Rochefort et al., 2011). This suggests a reliance on external cues, possibly to overcome an inability to properly use self-motion information alone. As a side note of interest, head direction cells in both the retrosplenial cortex and the anterodorsal nucleus of the thalamus similarly are more unstable in the dark in L7-PCKI mice than WT mice, but will anchor appropriately to an external cue in light conditions (Fallahnezhad et al., 2021), suggesting parallel deficits in other components of the navigational system.

Cue rotation allows further investigation of the L7-PKCI phenotype (Figure 5H). When a hidden rotation occurs, both WT and L7-PKCI place cells will remap, rotating with the cue, indicating a reliance on the current location of the cue. Things get interesting when the animals are able to view the cue’s rotation. WT animals appear to realize that the cue has been relocated, and their place cells do not remap (i.e., do not follow the object cue). L7-PKCI animals’ place cells, on the other hand, show various levels of remapping, as if strongly driven by the cue and unable to resolve the mismatch (30% of place fields remained stable; 20% exhibited a rotation of ∼180 degrees, matching the 180 degree cue rotation; and 50% remapped at some other rotation angle) (Rochefort et al., 2011). This suggests a weaker use of self-motion information, and a greater reliance on environmental cues in L7-PCKI mice.

Even without environmental perturbations, a smaller proportion of hippocampal cells are identified as place cells in L7-PKCI mice (Rochefort et al., 2011), potentially indicating deficits in hippocampal spatial processing even in the absence of overt behavioral deficits. That is, even when the hippocampus has sufficient information to form spatial maps, insufficient self-motion (or other) information from the cerebellum may weaken that neuronal representation.

In many ways, a mirror picture emerges when considering L7-PP2B mice. Even in the light, with an anchoring cue present, these mice sometimes have unstable place cells (Lefort et al., 2019). Specifically, when place cells are unstable (approximately 25% of the sessions), they appeared to be unstable together, rotating as an ensemble (Figure 5I). It is worth noting that this instability happens between sessions, not within a session. Remapping occurs as a ‘coherent rotation of the whole spatial representation’ (Lefort et al., 2019) – as if the hippocampus believes the arena has been rotated when it has not – suggesting insufficient use of the anchoring cue in determining orientation. However, it was not that the animals were simply disinterested in the object, as when these field rotations occurred, L7-PP2B animals showed an increased exploration of the cue (Lefort et al., 2019). This is fitting with the animals believing the cue has been moved, as animals generally spend increased time investigating objects in novel locations (Vogel-Ciernia and Wood, 2014). Similarly, under light conditions, head direction cells in the anterodorsal thalamus in L7-PP2B mice were less stable than controls (approximately as stable as they were in the dark) suggesting a lack of use of reference cues to anchor and maintain orientation (Fallahnezhad et al., 2021). Similarly, a 90-degree cue rotation results in an approximate 90-degree head direction rate map rotation in control animals but only about half that rotation in L7-PP2B mice, further suggesting incomplete use of anchor-cues in other components of the navigational system as well. As a final note of interest, no change in hippocampal theta or low gamma oscillations were noted in L7-PP2B mice, but there was an increase in hippocampal high gamma (Lefort et al., 2019).

Together with the L7-PKCI work, a picture emerges whereby animals with cerebellar plasticity deficits also show spatial navigation and hippocampal place cell deficits. More specifically, animals with impaired cerebellar LTD show phenotypes consistent with poor use of self-motion information (i.e., they need external objects to anchor) and animals with impaired cerebellar LTP show phenotypes suggestive of poor use of anchor objects.

**FIGURE 6:**
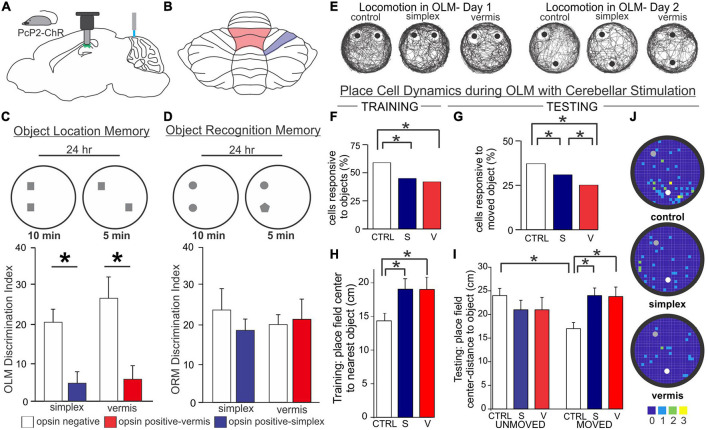
Acute cerebellar stimulation affects hippocampal-dependent object processing and place cells. **(A)** Experimental schematic: Mice expressing channelrhodopsin in Purkinje cells (PcP2-ChR) were used throughout and received blue light stimulation once per minute during all behavior. Miniscopes [used in experiments presented in **(E-J)**] were placed above the hippocampus and used to image calcium activity in CA1. **(B)** Schematic highlighting lobule IV/V vermis (red outline) and simplex (blue outline) – the cerebellar regions targeted in the experiments presented in this figure. **(C,D)** In a hippocampal-dependent Object Location Memory (OLM) task and a largely hippocampal-independent Object Recognition Memory (ORM) task, animals were placed into an arena with two objects and allowed to explore for 10 min. 24 h later, animals were re-placed into the arena but one object was relocated (OLM) or exchanged for another object (ORM). The discrimination index represents the relative time spent exploring the object with spatial or identity novelty. Manipulation of the cerebellum caused a marked impairment on the OLM task **(C)** but not the ORM task **(D)**, suggesting a specific deficit in processing objects in space, rather than a motor or other general deficit. **(E)** Location tracking map in an OLM arena showing the animals’ trajectory during each day. Gross movement was not affected by cerebellar stimulation as all groups thoroughly explored the arena. **(F,G)** Cerebellar stimulation resulted in a reduced responsiveness of CA1 cells to objects in the arena on both days. CA1 place fields mapped closer to objects in control animals during training **(H)** and closer to the moved object during testing **(I)**. This was disrupted in animals with stimulation of the vermis or simplex. **(J)** Place field centers on Day 2. Note the clustering of place cells near the moved (white dot) object compared to the unmoved object (gray dot) in control, but not cerebellar stimulated, animals. Color bar represents number of place fields in each bin across animals. Figure panels **(C-J)** modified from (Zeidler et al., 2020). V, vermis; S, simplex; CTRL, control.

While these studies examining L7-PKCI and L7-PP2B mice rely on genetic manipulations of plasticity, which have chronic and developmental impacts potentially at play, a similar finding of altered hippocampal processing of objects in space has been found with acute cerebellar manipulation. Specifically, Zeidler et al. found that periodic optogenetic stimulation of Purkinje cells (in either the simplex or lobule IV/V of the vermis) results in impaired processing of objects in space (Figure 6; Zeidler et al., 2020). During initial presentation of objects, fewer hippocampal cells were responsive to objects in the arena when Purkinje cells were manipulated. Place cells will often anchor fields near objects (Manns and Eichenbaum, 2009; Burke et al., 2011), and this was impaired in animals receiving Purkinje cell stimulation (Zeidler et al., 2020). When one of the objects was later displaced, control animals showed an increase in place cells near that object, an effect that was absent in animals with Purkinje cell stimulation (Figure 6). Corresponding to this hippocampal processing impairment, animals receiving Purkinje cell stimulation showed a behavioral impairment on an object location memory task that is largely hippocampal-dependent (Barker and Warburton, 2011; Haettig et al., 2013) compared to control animals that spent increased time investigating the object in a novel location (Figure 6). Importantly, this deficit could not be attributed to impaired motor function, as similar amounts of total time were spent investigating objects overall. There was also no deficit on a similarly structured, non-spatial, and largely hippocampal-independent task for object recognition memory (Figure 6; Balderas et al., 2008; Barker and Warburton, 2011; Vogel-Ciernia and Wood, 2014). This highlights that the effect of cerebellar manipulation created a specific deficit regarding the processing of objects in space, rather than all object processing in general.

**FIGURE 7:**
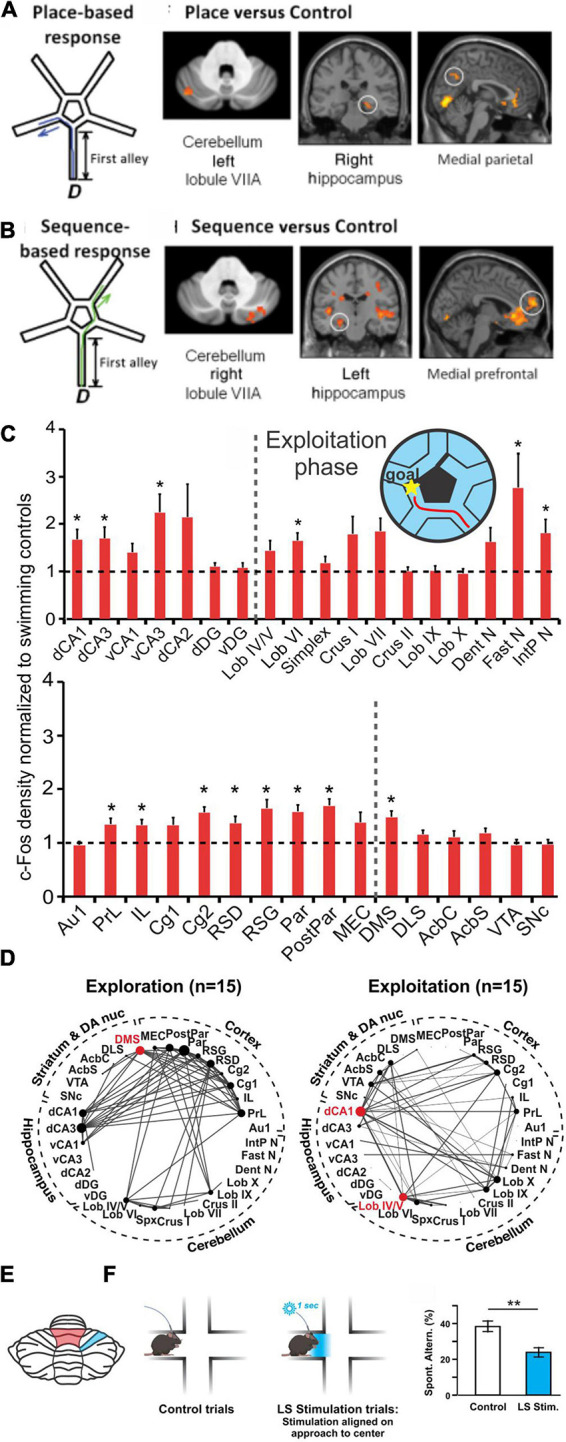
Cerebello-hippocampal interactions during navigation. **(A,B)** Human participants underwent a virtual reality navigation task, and their navigation strategies were categorized as place-based **(A)** or sequence-based **(B)**. In the allocentric, anchoring-cue, place-based strategy (schematic in **A**), participants memorized turns needed to get to the goal based on environmental cues. In contrast, the egocentric, self-motion, sequence-based strategy (schematic in **B**) relied on reproducing body turns in a correct sequence. Regardless of search strategy used, the cerebellum (VIIA) and the hippocampus were co-activated. However, place-based strategies **(A)** resulted in *left* cerebellum being co-activated with the right hippocampus (and medial parietal), while motion-based strategies **(B)** resulted in *right* cerebellum being co-activated with left hippocampus (and medial prefrontal cortex). **(C)** Mice completed a navigation task in which they learned to swim towards a goal (maze design pictured in inset). cFOS labeling was measured and normalized to swimming-only controls (dotted line shows normalization) in both animals that had not yet learned an efficient route towards the goal (termed “exploration phase”; not pictured) and animals that had learned to complete the task efficiently (termed “exploitation phase”; red line in inset maze and cFOS bar graphs). cFOS expression was significantly increased in the exploitation phase in regions including the hippocampus and cerebellum. **(D)** Interregional correlation analyses were performed based on cFOS results and network graphs were made based on Spearman correlations stronger than 0.64. Comparison of exploration (left) and exploitation (right) phases of the task revealed a shift of network hub structures (red dots) from the dorsomedial striatum (DMS) to dorsal CA1 and vermal lobule (Lob) IV/V (red shading in the schematic in **E**) once the task was learned and completed efficiently. **(E)** Regions in the cerebellum shown to be collaborating with the hippocampus (in **D**) or affecting navigational efficiency (in F) during navigation in rodents. **(F)** During a spontaneous alternation task that tests spatial working memory (SWM) (Lalonde, 2002), mice that received optogenetic activation of the simplex lobule (LS; blue shaded region in panel E) had lower levels of spontaneous alternation than control mice (Liu et al., 2022). Taken together, data from mice and humans shows a collaboration of the cerebellum and the hippocampus during spatial navigation. Panels A&B (data and schematics) are reproduced from Iglói et al. (2015) with permission from Oxford University Press. Data in panels **(C,D)** were adapted and reformatted for arrangement in this review from Babayan et al. (2017) under a CC-BY 4.0 license. Panel **(F)** (data and schematic) was reproduced from Liu et al., 2022 with permission from Springer. CA1, dorsal (dCA1) and ventral (dCA1); CA3, dorsal (dCA3) and ventral (vCA3); dorsal CA2 (dCA2); dentate gyrus, dorsal (dDG) and ventral (vDG); simplex (spx); dentate nucleus (Dent N); fastigial nucleus (Fast N); interposed nucleus (IntP N); cortex, primary auditory (Au1), prelimbic (PrL), infralimbic (IL), cingulate (Cg1,Cg2); retrosplenial, dysgranular (RSD) and granular (RSG); parietal (Par) and posterior parietal (PostPar); medial entorhinal (MEC); dorsolateral striatum (DLS); nucleus accumbens core (AcbC) and shell (AcbS); ventral tegmental area (VTA); substantia nigra pars compacta (SNc).

Optogenetic manipulation of the simplex may also disrupt sequence-based navigation. Specifically, optogenetic stimulation of the lateral simplex of the cerebellum when the mouse reached the center of the split in a plus maze resulted in lowered levels of spontaneous alternation (Figure 7; Liu et al., 2022). Interestingly, a decrease in coherence between the medial PFC and the dorsal hippocampus at multiple frequency bands was also observed with this manipulation (Liu et al., 2022).

Taken together, both chronic and acute cerebellar manipulations illustrate that disrupted cerebellar processing can alter hippocampal function, with impairments able to impact both self-motion processing and object-based anchoring, sometimes (in the case of L7-PKCI vs L7-PP2B) differentially (Burguière et al., 2005; Rochefort et al., 2011; Lefort et al., 2019; Zeidler et al., 2020).

Human imaging work also suggests collaboration between the hippocampus and the cerebellum during spatial navigation, whether tracking self-motion or instead primarily using anchoring cues. This is beautifully illustrated in a study which had participants complete a maze in virtual reality while in an fMRI scanner (Iglói et al., 2015). Participants navigated to a goal and researchers then analyzed their paths to determine whether they used a “sequence-based” (self-motion) or “place based” (anchor-cues) strategy, reaching their goal either by completing the same turns as in training or by using location cues in the environment, respectively (Figures 7A, B; Iglói et al., 2015). With either strategy, the cerebellum (Crus I) and hippocampus were coactivated (Figures 7A, B). While lateralization may be less likely in the rodent studies we have previously discussed, an interesting lateralization finding occurred when this human-based data was analyzed by navigational strategy. Specifically, right cerebellar Crus I was coactivated with the left hippocampus (and mPFC) in motion based strategies, and the left Crus I was coactivated with the right hippocampus (and medial parietal cortex) during anchor-cue based navigation (Iglói et al., 2015). Overall, this work suggests that the hippocampus and the cerebellum are working together in spatial working memory, though their collaboration may depend on strategy.

Recently, circuits underlying sequence (self-motion) based navigation were examined in rodents using cFos exploring a cue-impoverished environment (to force a self-motion strategy) (Figure 7C; Babayan et al., 2017). Animals that successfully learned an optimal route to the escape platform had increased cFos in a number of areas, including both the cerebellum and the hippocampus (Figure 7C). While increased cFos expression happened in both areas regardless of ability to routinely find an optimal path, inter-regional correlation analysis showed an importance difference. Animals that were able to exploit their knowledge (i.e., were able to use motion sequence-based navigation efficiently to reach the escape platform), showed increases in covariance of cFos expression (indicative of functionally connected networks) between the cerebellum and hippocampus (Figure 7D). In particular, lobule IV/V showed high connectivity with CA1 (Figure 7D; Babayan et al., 2017). Interestingly, lobule IV/V has been shown to be able acutely influence CA1 (Figures 2A–C; Zeidler et al., 2020) but was not an area highly labeled after rabies injection into the dentate gyrus (Figure 1F; Watson et al., 2019). Combined with the findings of specifically strong increases in coordinated activity in this task between lobule IV/V and CA1 (Babayan et al., 2017), this raises the interesting possibility of additional cerebellar-CA1 connectivity not captured by the initial rabies tracing work.

The studies presented in this section show several important facets of the cerebellum’s role in spatial navigation:

1) Its role cannot solely be attributed to motor functions.
2) Either acute or chronic cerebellar disruption can alter hippocampal spatial processing and disrupt place cell representations.
3) Proper cerebellar functioning appears to be important for the hippocampus to use data about both egocentric (self-motion) and allocentric (object localization) information.

In short, Hippobellum coordination appears to be a critical component in spatial navigation.

## The social Hippobellum?

Both the cerebellum (Stoodley and Schmahmann, 2009; Hoche et al., 2016; Badura et al., 2018; Guell et al., 2018; Van Overwalle et al., 2020) and the hippocampus (Kogan et al., 2000; Okuyama et al., 2016; Sliwa et al., 2016; Chiang et al., 2018) are involved in social processing. The earliest evidence for cerebellar involvement in social processes comes from the characterization of Cerebellar Cognitive Affective Syndrome (CCAS), a disorder in which patients display deficits in affective processing (Schmahmann and Sherman, 1998). A meta-analysis of functional studies in humans pointed to cerebellar involvement in abstract forms of social processing in particular, such as mentalizing and mirroring others’ mind and body states during social interactions (Van Overwalle et al., 2014), and, indeed, patients with cerebellar disorders struggle with these abstractions (Hoche et al., 2016). In mice, manipulation of cerebellar circuitry results in altered social behavior (Badura et al., 2018; Locke et al., 2018, 2020; Carta et al., 2019; Chao et al., 2020b,^2021^). Human fMRI studies indicate several cerebellar domains may be involved in social processing, including lobule VI/Crus I, lobule VIIb/Crus II, and lobules IX/X (Stoodley and Schmahmann, 2009; Guell et al., 2018).

**FIGURE 8:**
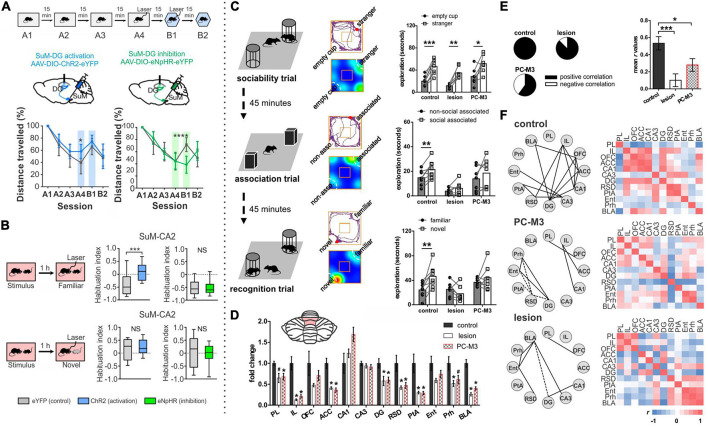
Potential for cerebellar-hippocampal interactions in social processing. **(A,B)** The supramammillary area (SuM) is a potential relay station between the cerebellum and the hippocampus, as it projects to the hippocampus via two separate anatomical pathways, and receives direct inputs from the cerebellum. **(A)** Activation or inhibition of SuM-DG projections alters spatial novelty processing. Contextual behavior tasks were conducted with three habituation sessions (A1-A3) in a high-walled enclosure (top schematic). Distance traveled decreased across the habituation sessions suggesting familiarization with the environment (bottom plots). Light was delivered to the DG in sessions A4 and B1, to activate (blue lines and shading) or inhibit (green lines and shading) local SuM terminals. Gray lines represent opsin-negative controls. Activation of SuM-DG terminals increased the distance traveled in the A4 session (left bottom), indicating exploration associated with increased perception of novelty. In session B1, the chamber was altered. Inhibition of SuM-DG neurons prevented recognition of this contextual novelty, as indicated by a lack of increase in distance traveled in the B1 session (right bottom). **(B)** Activation of SuM-CA2 terminals alters social novelty processing. Mice were first exposed to a novel mouse without light delivery, and then one hour later mice were either exposed to the same mouse (testing recognition, top schematic) or a novel mouse (bottom schematic) concurrently with light delivery. Activation of SuM-CA2 neurons resulted in increased social interaction when re-exposed to the same mouse, as if the mouse were novel (top, middle). No effects were seen on time spent exploring a novel mouse, or with optogenetic inhibition, rather than excitation, of SuM-CA2 terminals. **(C–F)** Cerebellar manipulations – via either neurotoxic lesion or chemogenetic excitation of Purkinje cells (PC-M3) in cerebellar lobules IV/V – impact social memory, but not general sociability, and fragment hippocampal connectivity patterns. **(C)** Left: Three trials tested sociability (exploration of a stranger mouse and an empty cup; top), animal-location association (exploration of two identical objects, with one in the location of prior mouse; middle), and recognition (exploration of familiar and novel stranger; bottom). All groups had intact sociability behavior and spent more time exploring the mouse compared to an empty container (top right). In contrast, only the control group had intact social-associative memory (middle right) and social recognition (middle bottom). **(D)** Lesion or chemogenetic manipulation of lobule IV/V reduced c-Fos expression after free social interaction in various brain regions including the hippocampal dentate gyrus (DG). Inset: rodent flatmap showing the location of cerebellar manipulations in lobule IV/V. **(E)** Cerebellar manipulation resulted in relatively more negative correlations between brain regions and in a lower average correlation overall (r) following a free social interaction task. **(F)** Detailed interregional correlations for c-Fos expression following a free social interaction task. Solid lines represent positive correlations and dashed lines represent negative correlations. Note the reduced connectivity of hippocampal regions (DG, CA3, CA1) with other brain regions in animals with cerebellar manipulation. Panels **(A,B)** are modified slightly and reproduced from Chen et al. (2020) with permission from Springer. Panels C-F modified slightly and reproduced from Chao et al. (2021) with permission from Springer. SuM, supramammillary area of the hypothalamus; DG, dentate gyrus; CA2, hippocampal CA2; PL, prelimbic cortex; IL, infralimbic cortex; OFC, orbitofrontal cortex; ACC, anterior cingulate cortex; DG, dentate gyrus; RSD, dorsal retrosplenial cortex; PtA, parietal association cortex; Ent, entorhinal cortex; Prh, perirhinal cortex; BLA, basolateral amygdala.

While multiple hippocampal subregions are associated with social processing (Hitti and Siegelbaum, 2014; Okuyama et al., 2016; Smith et al., 2016; Chiang et al., 2018; Meira et al., 2018), hippocampal CA2 – a region long neglected but finally receiving due attention (Caruana et al., 2012; Dudek et al., 2016) – may be especially interesting to consider. Dorsal CA2 pyramidal neurons project to and excite ventral CA1 pyramidal cells in support of social memory (Meira et al., 2018), lesions to CA2 cause impairments in social discrimination and recognition (Stevenson and Caldwell, 2014), and inhibition of either CA2 pyramidal neurons broadly (Hitti and Siegelbaum, 2014) or of a specific subpopulation expressing the vasopressin1b receptor (Smith et al., 2016) leads to deficits in social recognition memory. As noted previously, the SuM has projections to the hippocampus (Wyss et al., 1979; Haglund et al., 1984; Pan and McNaughton, 2004; Hashimotodani et al., 2018; Chen et al., 2020). These SuM projections are largely segregated, with dual glutamatergic-GABAergic inputs to the dorsal dentate gyrus (Hashimotodani et al., 2018) believed to be important for spatial novelty signals (Chen et al., 2020; Li et al., 2020) and glutamatergic inputs to the dorsal CA2 (and ventral dentate gyrus) important for social novelty signals (Figures 8A, B; Chen et al., 2020). Therefore, the SuM could potentially provide two independent channels by which the cerebellum could influence both spatial (via SuM projections to the dorsal dentate gyrus) and social (via SuM projections to the CA2) hippocampal functions. However, as noted above, work by DuLac’s group suggests a lack of a disynaptic fastigial to hippocampus projection (Fujita et al., 2020), and the SuM may instead route its influence on the hippocampus via e.g., the septum (Pan and McNaughton, 2004; Griguoli and Pimpinella, 2022).

There are of course many other potential anatomical substrates to support hippobellum interactions during social tasks, including DCN connections with the VTA that show increased activity during social interactions (Carta et al., 2019), the mediodorsal thalamus (Middleton and Strick, 1998; Rogers et al., 2013; Fujita et al., 2020; Judd et al., 2021; Streng et al., 2021), and/or the ventromedial thalamus (Aumann et al., 1994; Ichinohe et al., 2000; Kelly et al., 2020; Judd et al., 2021). These areas could potentially influence the hippocampus via the medial prefrontal cortex (Cenquizca and Swanson, 2007; Rajasethupathy et al., 2015; Malik et al., 2022), a structure also heavily involved in social cognition (recently reviewed in Bicks et al., 2015).

Interestingly, disruption of connectivity between the mPFC and the cerebellum (in particular Crus I) is reported for both autism models and individuals with autism (Kelly et al., 2020). Indeed, autism provides an additional context to explore the hippocampus and cerebellum in social processes, as there are structural and functional alterations of both the cerebellum and the hippocampus in autism (Banker et al., 2021; Mapelli et al., 2022). Additionally, animal models of autism often display cerebellar dysfunction, hippocampal dysfunction, or both (Brielmaier et al., 2012; Napoli et al., 2012; Varghese et al., 2017; Modi et al., 2019). For example, in the cerebellum of the Neuroligin3 Knockout model, mice have altered development and physiology in conjunction with altered overt social behaviors (Baudouin et al., 2012; Lai et al., 2021). In addition, there are changes in oscillatory activity and increased excitability in CA2 pyramidal neurons in the hippocampus (Modi et al., 2019).

While there is mounting research regarding the hippocampus and the cerebellum (separately) in social processing, very little research has looked at the potential for coordinated efforts of these two structures in social processing. One notable exception is work by Chao and colleagues examining cFos expression, including in the hippocampus, after cerebellar manipulations following a free social interaction task (Chao et al., 2021). Specifically, Chao and colleagues either lesioned or chemogenetically activated Purkinje cells in the cerebellar vermis (lobule IV/V) and found deficits in social processing (Figure 8C). Specifically, while sociability (preference for a novel mouse over an empty cup) was intact, social spatial association/memory and social recognition were impaired. While lesions also produced motor deficits, which may complicate interpretations, animals which had chemogenetic activation of Purkinje cells did not, suggesting true impacts on social processing. Importantly in the context of the hippobellum, Chao and colleagues found decreased cFos expression in the dentate gyrus and a fragmentation of interregional activation patterns with either lesion or chemogenetic Purkinje cell activation following free social interaction (Figures 8D–F). It is also interesting that this Purkinje cell manipulation affects social recognition, which is at least in part mediated by hippocampal subregions as noted previously (Hitti and Siegelbaum, 2014; Stevenson and Caldwell, 2014; Smith et al., 2016; Meira et al., 2018). Together, these data suggest a potential influence of the cerebellum on the hippocampus in social processing.

In summary, both the cerebellum and the hippocampus are involved in social processes, and current research hints at potential interactions between these structures during social behavior and in the context of autism, but much more work is needed to investigate the potential for collaboration between these structures for social processing along with any associated circuitry.

## The cerebellum and temporal lobe epilepsy

**FIGURE 9:**
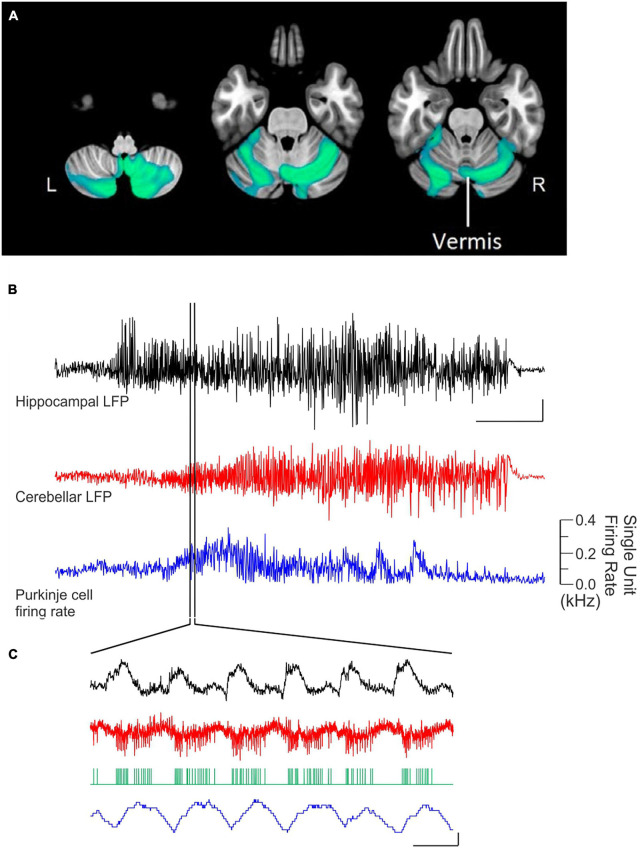
Seizures impact the cerebellum. **(A)** Epilepsy patients that later died from a SUDEP event showed gray matter volume loss (green/blue overlay) in the cerebellum compared to controls. This not only indicates that the cerebellum can be impacted by epilepsy but also that the cerebellum may be critical to outcomes in epilepsy. **(B)** Electrographic recordings illustrate acute impacts of spontaneous hippocampal seizures on the cerebellum. Shown in black is a seizure recorded from the hippocampus in a mouse model of temporal lobe epilepsy and the corresponding cerebellar local field potential (LFP) in red. The firing rate of an individual Purkinje cell during this seizure is shown in blue. The seizure was visible in the cerebellum prior to any overt behavioral component of the seizure, making a movement explanation unlikely. **(C)** The region indicated in **(B)**, on an expanded time scale, allowing visualization of the time-locked firing of the Purkinje cell (red trace) to the hippocampal ictal spiking. Green ticks represent detected spikes. Scale bars: B top: 1 mV, 10 s; B middle: 0.5 mV; C: 0.5 mV, 0.1 kHz change, & 0.1 s. Panel A reproduced from Allen et al. (2019) under a CC-BY 4.0 license. Panels **(B,C)** reproduced with slight modification from Krook-Magnuson et al. (2014).

Temporal lobe epilepsy (TLE) involves seizures typically arising from the hippocampus and is one of the most prevalent forms of epilepsy (Asadi-Pooya et al., 2017). TLE is often treatment resistant (Laxer et al., 2014; Tian, 2018), and there is a great need to better understand and treat the disorder. While hippocampal sclerosis is hallmark feature of TLE (Bouilleret et al., 1999), structural changes are not restricted to the temporal lobe, especially as the disease progresses (Sandok et al., 2000; Ibdali et al., 2021). The cerebellum in particular has been shown to be vulnerable in epilepsy, showing cell loss and atrophy (Norman, 1964; Crooks et al., 2000). One recent systematic review found three consistent predictors of cerebellar degeneration in epilepsy: i) phenytoin use as an anti-seizure drug, ii) poor seizure control, and iii) temporal lobe epilepsy (Ibdali et al., 2021), with the latter finding suggesting the cerebellum may be particularly impacted by hippocampal seizures. Sandok et al. found that cerebellar atrophy was bilateral and associated with the duration of TLE at the time of imaging (Sandok et al., 2000; but see also Marcián et al., 2018). Recent work also suggests that cerebellar atrophy may be critically important to outcomes in TLE, especially as it relates to SUDEP: sudden unexpected death in epilepsy. Specifically, Diehl and colleagues retrospectively examined anatomical images of patients that had later succumbed to a SUDEP event and compared gray matter volumes between patients deemed to be at a high risk for SUDEP, patients deemed to be at low risk for SUDEP, and healthy controls (Allen et al., 2019). Patients that went on to experience a SUDEP event showed strong bilateral cerebellar volume loss, including both vermal and lateral portions (Figure 9A; Allen et al., 2019). High risk patients also showed cerebellar volume loss relative to controls, but this was limited to the vermis. Notably, cerebellar loss in these groups remained even after removing patients with a history of phenytoin use (Allen et al., 2019), a drug which is known on its own to be related to cerebellar atrophy (Kessler et al., 1985; Botez et al., 1988; Ibdali et al., 2021). These findings strongly underscore the significance of cerebellar changes in temporal lobe epilepsy, especially when considering the magnitude of lives lost due to SUDEP: it is estimated that there are 1.16 cases of SUDEP per 1,000 people diagnosed with epilepsy, equating to over 100,000 years of potential life lost in the United States (Thurman et al., 2014).

Beyond overt changes to cerebellar structure that can occur with TLE progression, acute changes in cerebellar activity can be detected during hippocampal seizures. This was noted in early studies in which cobalt was injected into the hippocampus to create a seizure focus – the cerebellum was one of the first brain regions where epileptiform activity spread in cats (Babb et al., 1974) and in monkeys (Heath, 1976). Epileptiform-induced alterations in Purkinje cell firing was also noted with electrically and/or penicillin evoked hippocampal after discharges (Mitra and Snider, 1975; Heath et al., 1980). More recently, changes in Purkinje cell firing rates and local field potentials were noted in spontaneous seizures arising from the hippocampus in a mouse model of TLE (Figures 9B, C; Krook-Magnuson et al., 2014). This occurred even without overt motor manifestations of the seizures, making a motor explanation unlikely. Finally, in human patients, there are blood flow changes in the cerebellum during temporal lobe seizures (Won et al., 1996; Shin et al., 2001; Weder et al., 2006; Blumenfeld et al., 2009; Han et al., 2016).

**FIGURE 10:**
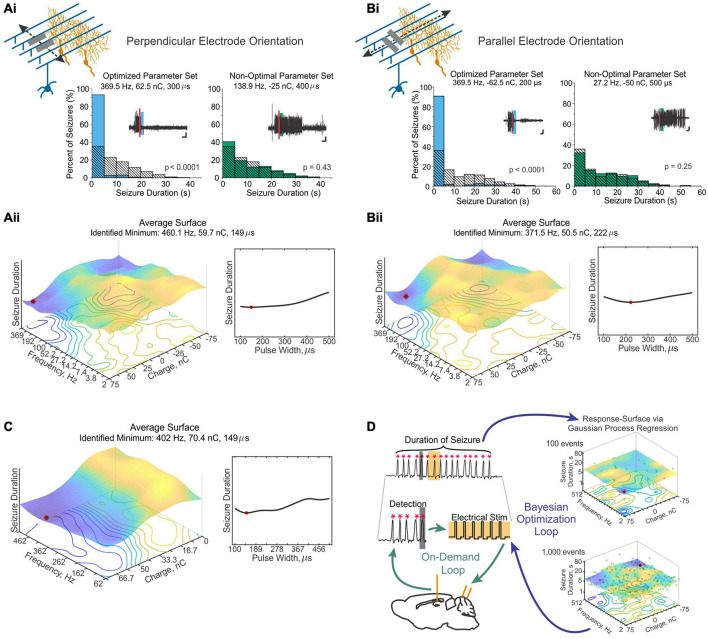
Optimization allows identification of settings that provide robust inhibition of hippocampal seizures via cerebellar electrical stimulation. Electrical stimulation was applied to the vermis, and similar results were achieved whether the stimulating electrodes were oriented perpendicular to the midline **(A)** or parallel to the midline **(B)**. **(Ai,Bi)** With optimized settings (left graph; blue bars) seizures were inhibited. No seizure inhibition was achieved when settings predicated to be ineffective from the optimization phase were instead used (right graph; green bars). Hashed bars represent no-stimulation control seizures. Histograms represent seizure duration distributions from example animals. Insets illustrate example seizures receiving each type of intervention; the vertical red line indicates the time of seizure detection; the green and blue bars indicate the time of electrical stimulation. **(Aii,Bii)** Average surface across animals, illustrating that low frequency or low charge stimulations were ineffective (warm colors), while high frequency (150-400 Hz) and strong charge (50-70 nC) inhibited seizures (cooler colors). Red dot represents the minimum of the surface. Given the high-dimensional nature of the data, only one cross section is shown. The colored plot shows the interaction of frequency and charge, given the pulse width associated with the minimum seizure duration. The impact of pulse width, at the ideal frequency and charge combination, is shown in a separate plot to the right. **(C)** An additional set of experiments further explored settings associated with seizure control. These data illustrate that there is a range of generally effective settings, and that the optimal setting is not the maximum charge and frequency. Therefore, while a strong charge and high frequency is optimal, it is not true that more/higher stimulation is better. **(D)** Schematic of the online optimization approach used to identify good settings and used to generate the curves presented in panels **(A-C)**. Seizures were detected online, triggering on-demand electrical stimulation (‘on-demand loop’). The effectiveness of the intervention (based on seizure duration) was also calculated online, and this was used to update a Gaussian process regression map of predicted outcomes. This was in turn used to select the next set of parameters to test, completing the second loop (‘Bayesian optimization loop’). This process allows an automatic, data-driven, approach to surface-mapping and parameter optimization. Scale bars: Ai: 5 s, 0.5 mV; Bi: 3 s, 0.33 mV. Panels reproduced and combined from 4 figures in Stieve et al. (2022) with permission from Oxford University Press.

Highlighting the bidirectional nature of the hippobellum, even in the context of epilepsy, the cerebellum is also able to impact hippocampal seizures. This is particularly exciting as new clinical treatment strategies are needed for TLE: in a significant percentage of cases, patients have intractable hippocampal seizures even after trying multiple different types and combinations of anti-seizure drugs (Tatum, 2012; Miterko et al., 2019). Stimulation of the cerebellum for seizure control is not a new idea and has been tested in both preclinical animal work and in small human trials (Miller, 1992; Norden and Blumenfeld, 2002; Fountas et al., 2010; Sprengers et al., 2017; Klinger and Mittal, 2018; Miterko et al., 2019; Streng and Krook-Magnuson, 2020b). However, results of studies using electrical stimulation of the cerebellum have been very mixed – some studies showed promising effects, but other studies showed no effect of electrical stimulation on seizures, or in some cases even a worsening of the epilepsy phenotype (recently reviewed in Streng and Krook-Magnuson, 2020b). Reasoning that the differences in outcome may have been in large part due to differences in stimulation parameters, our lab re-examined the potential for electrical stimulation of the cerebellum as a potential strategy to inhibit hippocampal seizures (Stieve et al., 2022). Given the large number of potential combinations to test, we turned to Bayesian optimization, allowing a data-driven approach to test over 1000 stimulation parameter combinations (Figure 10; Stieve et al., 2022). The results indicate that electrical stimulation of the cerebellum (targeting the vermis) can, indeed, consistently inhibit hippocampal seizures, and that the stimulation parameters chosen (but not the electrode orientation) are critically important to achieve this (Figures 10A, B). Specifically, a high frequency and strong charge (but not the highest or strongest tested) is needed for robust seizure inhibition (Figure 10C).

**FIGURE 11:**
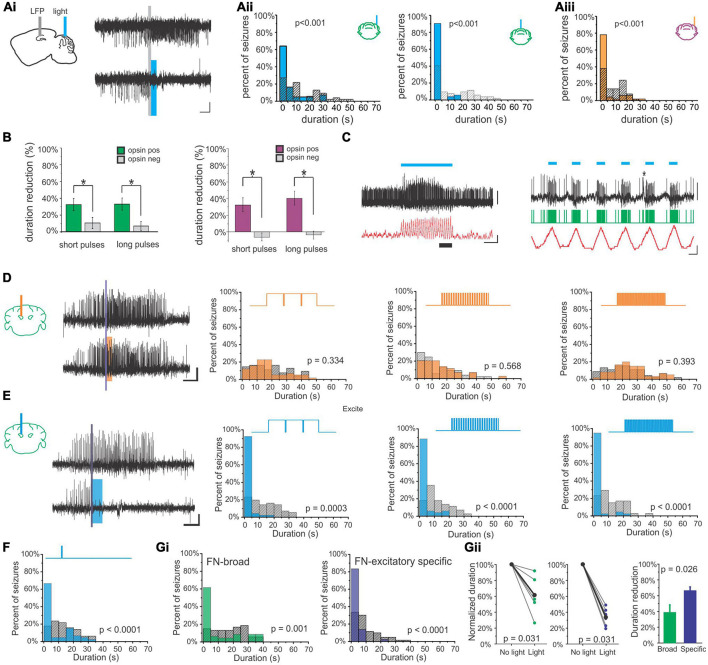
On-demand optogenetic manipulations of the cerebellum inhibit hippocampal seizures. **(A)** Seizures were detected in the hippocampus, triggering on-demand light delivery to the cerebellum. **(Ai)** An example hippocampal seizure detected (top; gray line indicates time of detection) but not receiving intervention (i.e., no-light internal control), and an example hippocampal seizure detected and receiving light delivery to the cerebellum (bottom; blue bar indicates timing of light delivery). Optogenetic manipulation of the cerebellar cortex inhibited hippocampal seizures whether light was targeted to the simplex (**Aii** left) or the vermis (**Aii** right), and whether exciting (**Aii**, blue bars) or inhibiting (**Aiii**, amber bars) Purkinje cells. Histograms represent post-detection seizure duration distributions from example animals. Colored bars represent events receiving light intervention, and hashed bars represent no-light internal controls throughout. **(B)** Optogenetic manipulation of the cerebellum was not strongly dependent on light-delivery parameters, with short pulses (50 ms on, 100 ms off) or long pulses (1,000 ms on, 50 ms off) effective for both optogenetic excitation (green bars) or inhibition (purple bars). Gray bars indicate data from opsin-negative control animals, receiving similar light pulses. Asterisks indicate significance at the *p* < 0.05 level. **(C)** Pulsed optogenetic excitation of Purkinje cells also resulted in pauses in Purkinje cell activity between light pulses; therefore, such light delivery may be somewhat similar to pulsed inhibition. Left: The black trace represents data from a juxtacellularly recorded Purkinje cell, with the blue bar indicating the period of pulsed light delivery. The red trace shows changes in firing rate over time. Right: an expanded view of the data presented to the left, from the time period indicated by the thick black bar at the bottom of the trace. Note the pauses in firing between light pulses. The asterisk indicates a detected complex spike. Green ticks represent detected spikes, and other colors are as described in left panel. **(D–G)** Hippocampal seizure control can also be achieved by targeting the fastigial nucleus, but excitation is required. **(D)** Optogenetic inhibition of fastigial neurons was unable to inhibit seizures, regardless of the stimulation parameters tried. The schematic above each histogram represents the light pulses delivered. **(E)** In contrast, optogenetic excitation of fastigial neurons provided strong seizure control. This was achieved regardless of stimulation parameters. **(F)** Even a single 50 ms light pulse delivered to the fastigial nucleus was able to inhibit hippocampal seizures. **(G)** While broad excitation of fastigial neurons (green) can inhibit hippocampal seizures, greater seizure control was actually achieved by restricting opsin expression to just excitatory cells (purple). Example animal histograms are shown in **(Gi)**, and summary data across animals is shown in **(Gii)**. Scale bars: A, D, E: 5 s, 0.05 mV; C left: 1 s; 1 mV or 100 Hz change in firing rate; C right: 50 ms; 1 mV or 100 Hz change in firing rate. Panels **(A–C)** modified from (Krook-Magnuson et al., 2014). Panels **(D–G)** modified from (Streng and Krook-Magnuson, 2020a).

The ability of the cerebellum to impact hippocampal seizures has also been seen in preclinical research using optogenetics, which can be especially valuable for understanding *how* the cerebellum is able to exert seizure-relieving effects on the hippocampus, such as what cell type(s) and output pathway(s) are involved. In on-demand optogenetic studies, online seizure detection allows light delivery to occur selectively at the very start of a seizure, allowing even greater experimental control and insight (Armstrong et al., 2013; Krook-Magnuson and Soltesz, 2015; Christenson Wick and Krook-Magnuson, 2018; Cela and Sjöström, 2019). On-demand optogenetic excitation or inhibition of Purkinje cells in the cerebellar vermis or the simplex is able to inhibit seizures in the intrahippocampal kainic acid mouse model of TLE (Figures 11A–C; Krook-Magnuson et al., 2014). Unlike electrical stimulation, this intervention is not particularly sensitive to stimulation parameters, with short or long pulses able to inhibit seizures (Krook-Magnuson et al., 2014). Surprisingly, downstream at the level of the FN, only excitation – but not inhibition – was able to inhibit seizures (Figures 11D–E; Streng and Krook-Magnuson, 2020a). This suggests that the seizure inhibition achieved with excitation of Purkinje cells (which would inhibit DCN neurons) may be due to the pauses in Purkinje cell firing seen immediately after excitatory light pulses (Figures 11A–C; Aizenman and Linden, 1999; Shin and De Schutter, 2006; Person and Raman, 2011; Dykstra et al., 2016). Regardless, excitation of fastigial neurons is required. When excitation of fastigial neurons is achieved, the precise light pulses are again not critical, with a range of stimulation frequencies effective (Figures 11D–F; Streng and Krook-Magnuson, 2020a). Interestingly, greater seizure control is achieved when only excitatory fastigial neurons are optogenetically targeted, rather than fastigial neurons broadly (Figure 11G; Streng and Krook-Magnuson, 2020a). This suggests that cell-type specificity of interventions may improve outcomes, and further highlights that excitation of excitatory fastigial neurons in particular is likely mediating seizure inhibition.

**FIGURE 12:**
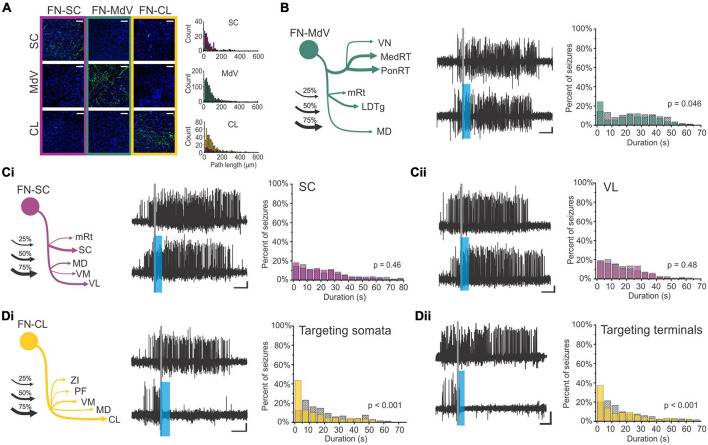
Fastigial neurons projecting to the central lateral thalamus are able to inhibit hippocampal seizures. **(A)** Fastigial outputs to the SC, MdV, and CL are via segregated channels, with little overlap. Shown are fibers in the SC, MdV, and CL after selective retrograde labeling of neurons based on their projections to the SC (FN-SC; magenta), the MdV (FN-MdV; teal), or CL (FN-CL; gold). Quantification of fibers in each region for each channel (color coded) is shown to the right. Note the general lack of overlap. **(B)** While these output channels are largely segregated, each does show collaterals to other areas (see also Figure 1). (left) The schematic shows key collaterals of FN neurons that were labeled based on their projection to the MdV (FN-MdV neurons), with line thickness indicating the relative strength of fiber labeling relative to broad-FN labeling. On-demand optogenetic excitation of FN-MdV neurons fails to provide strong seizure control. (middle) Example seizures detected (gray line), not receiving light (top; internal control) or receiving light to the fastigial to activate FN-MdV neurons. The blue bar indicates the time of light delivery. (right) Summary histogram across animals, showing poor seizure inhibition. Teal: events receiving optogenetic excitation of FN-MdV neurons. Hashed bars: no-light internal controls. A similar schema is applied throughout this figure. **(C)** FN neurons projecting to the SC have collaterals to other areas, including the VL thalamus. On-demand excitation of these cells, regardless of whether labeling these cells via their projections to the SC **(Ci)** or their projections to VL **(Cii)**, does not inhibit hippocampal seizures. **(D)** In contrast, on-demand excitation of FN-CL neurons **(Di)** or FN terminals in the CL (Dii) provides significant inhibition of hippocampal seizures. This suggests that a major route of influence of the cerebellum over the hippocampus, at least in the context of seizure control, is via the CL thalamus. Scale bars: A: 50 μm; B-D: 5 s, 0.05 mV. Panels modified from Streng et al. (2021). CL, centrolateral thalamic nucleus; LDTg, laterodorsal tegmental nucleus; MD, mediodorsal thalamic nucleus; MedRT, medullary reticular nucleus; MdV, medullary reticular nucleus, ventral; mRt, mesencephalic reticular formation; PF, parafascicular thalamic nucleus; PonRT, pontine reticular nucleus; SC, superior colliculus; VL, ventrolateral thalamic nucleus; VM, ventromedial thalamic nucleus; VN, vestibular nucleus; ZI, zona incerta.

As noted above, excitatory neurons in the fastigial nucleus project to over 60 different downstream regions (Figure 1; Fujita et al., 2020), making determining which output(s) is (are) important for seizure control difficult. However, the organization of fastigial outputs into output channels (wherein several downstream targets are collectively innervated by populations of fastigial neurons) provides some helpful structure. We investigated three such output channels: fastigial neurons projecting to the reticular formation (and other areas; Figures 1C, 12B), fastigial neurons projecting to the superior colliculus (in addition to other areas, including the ventral lateral thalamus; Figures 1C, 12C), and fastigial neurons projecting to the central lateral thalamus (in addition to other areas; Figures 1C, 12D; Streng et al., 2021). This was made possible by retrograde AAVs encoding Cre recombinase injected into the target area of interest, along with AAVs encoding the excitatory opsin channelrhodopsin in a Cre-dependent manner injected into the fastigial nucleus. Fastigial neurons which project to the reticular formation were unable to provide strong seizure control (Figure 12B; Streng et al., 2021). Fastigial outputs to the superior colliculus – whether targeted by their projections to the superior colliculus (Figure 12Ci) or when targeted by their projections to the ventral lateral thalamus (Figure 12Cii) – were also unable to inhibit seizures (Streng et al., 2021). However, selective on-demand optogenetic excitation of fastigial neurons projecting to the central lateral thalamus was able to successfully inhibit hippocampus seizures (Figure 12D; Streng et al., 2021). While broad excitation of fastigial neurons could produce small, overt, motor effects, no overt motor effects were seen when selectively activating fastigial neurons that project to the central lateral thalamus (Streng et al., 2021). Together, this work (i) identifies fastigial neurons that project to the central lateral thalamus (with collaterals to other areas) as playing a key role in cerebellar-mediated inhibition of hippocampal seizures, (ii) highlights that broad modulation of cerebellar outputs is not necessary to achieve seizure control, as one, specific, output channel was able to achieve significant seizure inhibition, and (iii) suggests a well-informed, restricted therapeutic approach providing sub-circuit specificity may be able to harness the benefits of cerebellar-directed interventions (i.e., seizure control) without some of the potential off-target effects associated with broad cerebellar manipulation (e.g., motor effects). A number of questions remain, including how these outputs are ultimately able to influence the hippocampus (the central lateral thalamus has no known connections to the hippocampus), if this same pathway is important in communication between the cerebellum and the hippocampus in healthy animals and under physiological conditions, and if other pathways may underlie additional aspects of cerebellar-mediated seizure inhibition. It should also be noted that the relationship between the cerebellum and the hippocampus in epilepsy is not unique; the cerebellum is also engaged by other types of seizures (Kandel and Buzsáki, 1993; Blumenfeld et al., 2009; Kros et al., 2015b,^2017^), shows structural changes (Botez et al., 1988; Crooks et al., 2000; Lee et al., 2018) and is a target of interest for seizure control (Kros et al., 2015b; Sprengers et al., 2017; Miterko et al., 2019) in other forms of epilepsy as well (Kros et al., 2015a; Streng and Krook-Magnuson, 2020b).

Overall, the relationship between the cerebellum and the hippocampus in TLE is an excellent example of the bidirectional communication at play in the hippobellum circuitry. Changes in the hippocampus during and as a result of epileptic events have acute and long-term effects on the cerebellum (which appear to have major impacts on clinical outcomes), and activating the cerebellum can powerfully inhibit hippocampal seizures.

## Conclusion

Interactions between the cerebellum and the hippocampus have been long underappreciated and difficult to study, but historical and emerging evidence strongly supports the existence and importance of bidirectional functional connectivity between these two structures. In order to fully understand hippobellum interactions, it is important to uncover the anatomical substrates underlying their communication. There is a growing body of research on cerebellar-hippocampal communications, and it promises new and exciting information for the field. Despite an incomplete picture of hippobellum interactions, they appear to be important in a range of contexts, from sleep to active exploration. Cerebellar-hippocampal interactions may also be important in areas not yet properly explored in the context of the hippobellum, including social processing. Their interactions can also be critically relevant in pathophysiology, including, most prominently, TLE. Indeed, the hippobellum may provide much needed potential new therapeutic avenues in TLE. These are early days for hippobellum research, and the future promises enlightening discoveries, providing critical insights into neuronal circuity and interregional communications.

## Author contributions

All authors have contributed to the drafting of the manuscript and read and approved the submitted version.
